# Relationship between rumination and symptoms of posttraumatic stress disorder – a cross-sectional network analysis

**DOI:** 10.3389/fpsyt.2026.1786768

**Published:** 2026-03-24

**Authors:** Olena Zhabenko, Ziv Ben-Zion, Paul Raffelhüschen, Achim Burrer, Erich Seifritz, Stefan Just, Katrin H. Preller, Or Duek, Megan Paterson, Robert H. Pietrzak, Jutta Joormann, Tobias R. Spiller, Ilan Harpaz-Rotem

**Affiliations:** 1 Department of Adult Psychiatry and Psychotherapy, University Hospital of Psychiatry Zurich (PUK), Zurich, Switzerland; 2 Department of Adult Psychiatry and Psychotherapy, University of Zurich (UZH), Zurich, Switzerland; 3 Department of Comparative Medicine, Yale University School of Medicine, New Haven, CT, United States; 4 Department of Psychiatry, Yale University School of Medicine, New Haven, CT, United States; 5 United States Department of Veterans Affairs National Center for PTSD, Clinical Neuroscience Division, Veterans Affairs (VA) Connecticut Health System, West Haven, CT, United States; 6 School of Public Health, Faculty of Social Welfare and Health Sciences, University of Haifa, Haifa, Israel; 7 Boehringer Ingelheim Pharma GmbH & Co. KG, Biberach an der Riss, Germany; 8 Boehringer Ingelheim (Schweiz) GmbH, Basel, Switzerland; 9 Department of Epidemiology, Biostatistics, and Community Health Sciences, Ben-Gurion University of the Negev, Beer-Sheva, Israel; 10 Department of Social and Behavioral Sciences, Yale School of Public Health, New Haven, CT, United States; 11 Department of Psychology, Yale University, New Haven, CT, United States; 12 Wu Tsai Institute, Yale University, New Haven, CT, United States

**Keywords:** anxiety, depression, network analysis, PTSD, rumination

## Abstract

**Background:**

Rumination is associated with the development and maintenance of PTSD. However, significant gaps remain in understanding how specific PTSD symptoms relate to rumination at a granular level, particularly when controlling for common comorbidities like depression and anxiety. This study used multi-level network analysis to examine these relationships in adult survivors of traumatic events.

**Methods:**

Six hundred sixty-one adult survivors (median age 35 years, 29 to 45 years, 49.3% females) who had witnessed or experienced a car crash, a violent act, or the killing of someone participated in an online-based study. Participants completed the Posttraumatic Stress Disorder Checklist (PCL-5), the Repetitive Thinking Questionnaire (RTQ-10), the Generalized Anxiety Disorder (GAD-7), and the Patient Health Questionnaire (PHQ-9). Network analysis was conducted at both scale and item levels to examine associations while controlling for depression and anxiety.

**Results:**

Network analysis revealed selective associations between rumination and symptoms of PTSD. At the cluster level, rumination was associated with re-experiencing and negative alterations in cognition and mood clusters, but not with avoidance or hyperarousal clusters. At the item level, complex patterns emerged including relevant associations between items assessing intrusive memories and persistent repetitive thoughts. Additionally, a negative association was observed between risk-taking behaviors and future-oriented wishful thinking.

**Conclusion:**

Our findings reveal interconnected constructs, which might in part be due to content overlap among the questionnaires used. These findings highlight the importance of precise construct definition and measurement differentiation when investigating trauma-related cognitive processes and establish groundwork for future research examining temporal relationships and clinical applications.

## 1 Introduction

Posttraumatic stress disorder (PTSD) represents a significant public health concern with far-reaching individual and societal implications. Beyond psychological distress, PTSD carries a substantial economic burden, with total costs in the United States alone estimated at $232.2 billion in 2018 (1). According to the DSM-5, PTSD encompasses four symptom clusters: intrusion symptoms (Cluster B), avoidance behaviors (Cluster C), negative alterations in cognition and mood (Cluster D), and alterations in arousal and reactivity (Cluster E) (2). Furthermore, PTSD frequently presents with comorbidities, most commonly depressive disorders, substance use disorders, and anxiety disorders, further complicating treatment approaches and recovery trajectories (3). Among the factors maintaining PTSD and its comorbidities, cognitive processes such as rumination have emerged as particularly important targets for understanding and intervention.

Rumination is a transdiagnostic cognitive process implicated in the development and maintenance of various psychopathologies (4). Rather than being disorder-specific in its content, rumination reflects a perseverative thinking style characterized by repetitive focus on negative themes, with the specific content varying depending on the clinical presentation (5). In the context of PTSD, rumination often manifests as perseveration over why the traumatic event occurred, counterfactual thinking about alternative outcomes, and repetitive analysis of trauma-related emotions and their consequences (6, 7). It has therefore been conceptualized as an unproductive coping mechanism that interferes with adaptive trauma processing and maintains psychological distress (8).

Accumulating evidence supports a relevant relationship between rumination and PTSD symptoms. A comprehensive systematic review encompassing 75 studies supported this association (9). However, the authors reported that the strength of this relationship varies across PTSD symptom clusters, indicating that a more granular examination is needed to better understand these interactions. For example, rumination has been found to more frequently trigger intrusive images in individuals with PTSD than in trauma-exposed controls, suggesting a direct mechanistic link between repetitive thinking and the re-experiencing symptom, but not other symptom clusters (10). Furthermore, the relationship between rumination and symptoms of PTSD is complicated by co-occurring depression and anxiety, which is highly prevalent among individuals with PTSD (11). Rumination was found to be heightened in individuals with comorbid PTSD and depression compared to those with depression alone, suggesting potential synergistic effects between these conditions (10). Additional research demonstrated that specific subtypes of rumination moderated the relationship between symptoms of PTSD and depression, underscoring the complex interplay between these psychological phenomena (12).

Despite this evidence, significant gaps limit our understanding of how rumination relates to specific symptoms of PTSD. First, most prior research has examined these constructs at sum score or symptom cluster levels, potentially obscuring important heterogeneity among individual symptoms (9, 10, 12). Cluster-level associations may be driven by only a few specific items, or conversely, weak cluster-level associations might mask strong connections between particular symptoms, and aspects of rumination. Without item-level examination, we cannot determine whether associations are distributed across symptom domains or concentrated in specific symptom-rumination pairings.

Furthermore, few studies have simultaneously controlled for comorbid depression and anxiety when investigating associations between PTSD and rumination (13–15). Thus, these results might be biased by the substantial shared variance among PTSD, depression, anxiety, and rumination reflecting both common underlying mechanisms (e.g., general distress or negative affect) as well as measurement overlap between instruments (16). Statistical approaches like network analysis can account for the shared variance of all variables simultaneously and can thus help identify unique associations between PTSD and rumination overcoming a common limitation of other approaches (17). Third, while intrusive memories are characterized by spontaneous, involuntary recall of traumatic content, rumination is more often characterized as a perseverative thinking process that is more deliberate (though not necessarily controllable) (6). Still, the conceptual boundaries between some PTSD symptom (e.g., intrusions) and rumination remain poorly delineated (16). Thus, distinguishing measurement artifact from genuine psychological overlap requires granular examination of item-level associations. Finally, understanding which specific PTSD symptoms are associated most strongly with rumination could inform treatment personalization (18). For instance, if rumination shows particularly relevant associations with avoidance symptoms, this might suggest that patients with prominent avoidance could benefit from rumination-focused cognitive interventions as adjuncts to exposure-based trauma therapy. However, such clinical applications require first establishing a detailed understanding of these symptom-level relationships.

Network analysis offers a methodological approach well-suited to address these gaps. By estimating partial correlations between symptoms, one can account for the shared variance of all other variables in the network, and thus identify specific pair-wise associations beyond shared variance (17–20). The resulting networks visualize symptoms or items as nodes, with edges representing the strength of unique associations between them after controlling for all other variables. Additionally, network analysis permits examination at multiple levels of granularity, allowing investigation of whether cluster-level associations reflect distributed connections across domains or are driven by specific item pairs (18). Previous network studies have demonstrated dense associations among symptoms of PTSD (21–23) between symptoms of PTSD and depression (24) as well as symptoms of generalized anxiety (25). Similar work focusing on rumination, found close interconnections between different aspects of rumination (26, 27). However, no studies have simultaneously examined PTSD symptom clusters and rumination while adjusting for the shared variance of the common comorbidities of depression and anxiety.

The current study used network analysis to examine associations between PTSD symptoms and rumination in a sample of trauma-exposed adults, while controlling for symptoms of depression and anxiety. We conducted analyses at two levels: (1) PTSD symptom clusters and overall rumination (assessed with the RTQ-10) to identify broad patterns of association, and (2) individual PTSD symptoms and specific RTQ-10 items to reveal granular connections and examine potential conceptual overlap between measurement instruments. This multi-level approach allows us to determine whether cluster-level associations are driven by specific symptom-item pairs or reflect more distributed relationships across domains. By accounting for depression and anxiety symptoms in all analyses, we aimed to isolate associations specific to PTSD and rumination beyond their shared variance with these common comorbidities, providing a more nuanced understanding of how rumination relates to the heterogeneous symptom presentation of PTSD. Without controlling for depression and anxiety, observed associations between rumination and PTSD symptoms could be confounded, as rumination is a transdiagnostic process also implicated in both depression and anxiety. This could lead to overestimating the direct relationship between rumination and PTSD, potentially misguiding intervention targets.

## 2 Methods

### 2.1 Participants and procedures

This research study used a cross-sectional online study design using the Qualtrics survey platform. Participants were recruited via crowdsourcing platform Prolific (28). Potential participants underwent pre-screening to ensure they were at least 18 years of age and had exposure to relevant trauma. The trauma exposure criteria were framed more restrictively than Criterion A of the DSM-5-TR definition and specifically included having witnessed or experienced a car crash, a violent act, or a homicide. This pre-screening approach was designed to ensure participants had experienced traumatic events likely to meet DSM-5-TR Criterion A requirements for PTSD, while maintaining feasibility for online data collection. If these initial criteria were met, participants were allowed to proceed to the screening phase of the study, which included the collection of demographic data, responses to the PTSD Checklist for DSM-5 (PCL-5) (29), and attention checks to ensure that the participants were human and attentive to the tasks. This screening phase was designed to ensure that reported data was of sufficient scientific quality. Participants were only allowed to proceed to the full assessment, including the assessment of all the measures reported below, when meeting all inclusion criteria, namely: being at least 18 years of age; successfully passing all attention checks; meeting Criterion A of the DSM-5-TR definition of PTSD; and completing all PCL-5 items. The study protocol received approval from the Yale University Institutional Review Board (IRB) but was not preregistered otherwise. This study does not have a clinical trial number. All procedures followed were in accordance with the ethical standards of the committee responsible for human experimentation (institutional and national) and with the Helsinki Declaration of 1975, as revised in 2000. All participants provided written informed consent. Additionally, participants were provided with resources for psychological support in case they experienced distress related to study content.

### 2.2 Measures

The collected demographic information included age, sex, and education level.

PTSD symptoms within the last month were assessed using the PTSD Checklist for DSM-5, with regard to the index trauma, the one bothering participants the most (29). This self-administered questionnaire consists of 20 items corresponding to the PTSD symptoms in the DSM-5, with each item scored from 0 (“not at all”) to 4 (“extremely”). The PCL-5 assesses four clusters of PTSD: Cluster B – intrusion symptoms (e.g., recurrent and involuntary memories, nightmares, and flashbacks); Cluster C – avoidance (e.g., places, people, feelings, and thoughts); Cluster D – negative alterations in mood and cognition (e.g., persistent negative beliefs and negative affect such as fear, anger, guilt, and shame); and Cluster E – hyperarousal (e.g., concentration and sleep problems, and hypervigilance). Total symptom severity scores range from 0 to 80.

Repetitive thinking was measured with the Repetitive Thinking Questionnaire 10-item (RTQ-10) (30), a shortened version of the RTQ-31 (31). This self-administered tool assesses worry, rumination, and anxiety/depression-related cognitions in relation to the index trauma using a 5-point Likert scale from 1 (“not at all”) to 5 (“very true”). The total score was summed for each participant, with a possible range from 10 to 50.

The Generalized Anxiety Disorder 7-item scale (GAD-7) (32) was used to assess anxiety levels. The scale evaluates symptoms over the past two weeks, with items scored from 0 (“not at all”) to 3 (“nearly every day”). Scores of 0–4 indicate minimal anxiety, 5–9 mild anxiety, 10–14 moderate anxiety, and scores above 15 severe anxiety.

Symptoms of depression were measured using the Patient Health Questionnaire 9-item (PHQ-9) (33) self-rating scale based on DSM-IV criteria. Each item is scored from 0 (“not at all”) to 3 (“nearly every day”), with total scores categorized as: 1–4 minimal depression, 5–9 mild, 10–14 moderate, 15–19 moderately severe, and 20–27 severe depression.

All measures demonstrated good internal consistency in this study: PCL-5 total (α = 0.89), PCL-5 re-experiencing cluster (α = 0.80), PCL-5 avoidance cluster (α = 0.73), PCL-5 negative cognitions cluster (α = 0.81), PCL-5 arousal cluster (α = 0.68), RTQ-10 (α = 0.93), GAD-7 (α = 0.86), and PHQ-9 (α = 0.89).

### 2.3 Data analysis

Demographic characteristics were summarized using numbers and percentages, or in case of continuous variables median and interquartile range (IQR). We used a network analytical approach to examine the complex connections between symptoms of PTSD, rumination, depression, and anxiety. Three distinct networks were estimated: a network of DSM-5 PTSD symptom clusters and rumination; a network of DSM-5 PTSD symptom clusters, rumination, levels of anxiety, and depression; and an item-level network of PTSD symptoms and rumination including levels of anxiety, and depression (to adjust for their influence). We examined zero-order correlations between all items to assess potential content overlap between measurement instruments. Following established practices in network analysis, items were retained if correlations did not exceed 0.5, indicating sufficiently distinct variance. Network structure was estimated using ggmModSelect relying on stepwise, unregularized Gaussian Graphical Models selection via the Extended Bayesian Information Criterion (EBIC) without assessing normality of the analyzed data (34). In contrast to regularized network estimation procedures, ggmModSelect does not assume network sparsity and thus is the best approximation of the true network structure (35). In the resulting Gaussian Graphical Model (GGM), nodes represent symptoms or questionnaire items, and edges represent associations between symptoms. Edge thickness and color indicate weight and direction (blue = positive, red = negative). We assessed network robustness and accuracy using a bootstrapping procedure implemented in the bootnet package (34). Due to the online study format, participants with missing data were excluded from subsequent data collection steps, resulting in complete-case analysis. All statistical analyses were performed in the *R* statistical environment.

## 3 Results

A total of 931 participants were eligible for the full study. Of there, six had missing demographic data, and further 273 did not fully complete the symptom questionnaires. Consequently, 661 individuals completed the study, of which 326 (49.3%) self-identified as female, 18 (2.7%) as non-binary or other, 4 (0.6%) preferred not to say, and the remaining 313 (47.4%) identified as male. Median age was 35 years (IQR 29-45), and median PCL-5 score was 33 (IQR 24-44) with 338 (51.1%) participants reporting a total PCL-5 score of 33 or higher indicating probable PTSD (29). Additional demographic information is presented in **Table 1**. Zero-order correlations between all RTQ-10 and PCL-5 items did not exceed 0.5. A full zero-order correlation matrix of all included symptoms, as well as the stability and reliability analyses, which revealed CS-coefficients exceeding 0.75 (equaling the largest value tested) for the first two networks and a CS-coefficient of 0.67 for the third network indicating stability, are shown in the **Supplementary**.

**Table 1:** Characteristics of the sample.

Variable	N	%/Median (IQR)
Age	661	35 (29-45)
Gender		
Male	313	47.4
Female	326	49.3
Non-Binary/Other	18	2.7
Prefer Not to Disclose	4	0.6
Education		
Less than High School	14	2.1
High School / GED	99	15.0
College (no degree)	168	25.4
Technical degree	76	11.5
BA	217	32.8
MA/PhD	85	12.9
Prefer Not to Disclose	2	0.3
PTSD (PCL-5) Score	661	33 (24-44)
Re-experiencing (B)	661	7.0 (5.0 – 11.0)
Avoidance (C)	661	4.00 (3.00 – 6.00)
Negative Cognitions (D)	661	12.0 (8.0 – 17.0)
Arousal (E)	661	9.0 (6.0 – 13.0)
Probable PTSD (PCL-5 ≥ 33)	661	338 (51.1)
Rumination (RTQ-10) Score	661	32 (24-39)
Anxiety (GAD-7) Score	661	9.0 (6.0-13.0)
Depression (PHQ-9) Score	661	11.0 (7.0-16.0)

IQR, Interquartile Range; GED, General Educational Development; BA, Bachelor of Arts Degree; MA, Master of Education; PhD, Doctor of Philosophy, research degree; PTSD, Posttraumatic Stress Disorder; PCL-5, PTSD Checklist for DSM-5; RTQ-10, Repetitive Thinking Questionnaire; GAD-7, Generalized Anxiety Disorder; PHQ-9, Patient HealthQuestionnaire.

**Figure 1:**
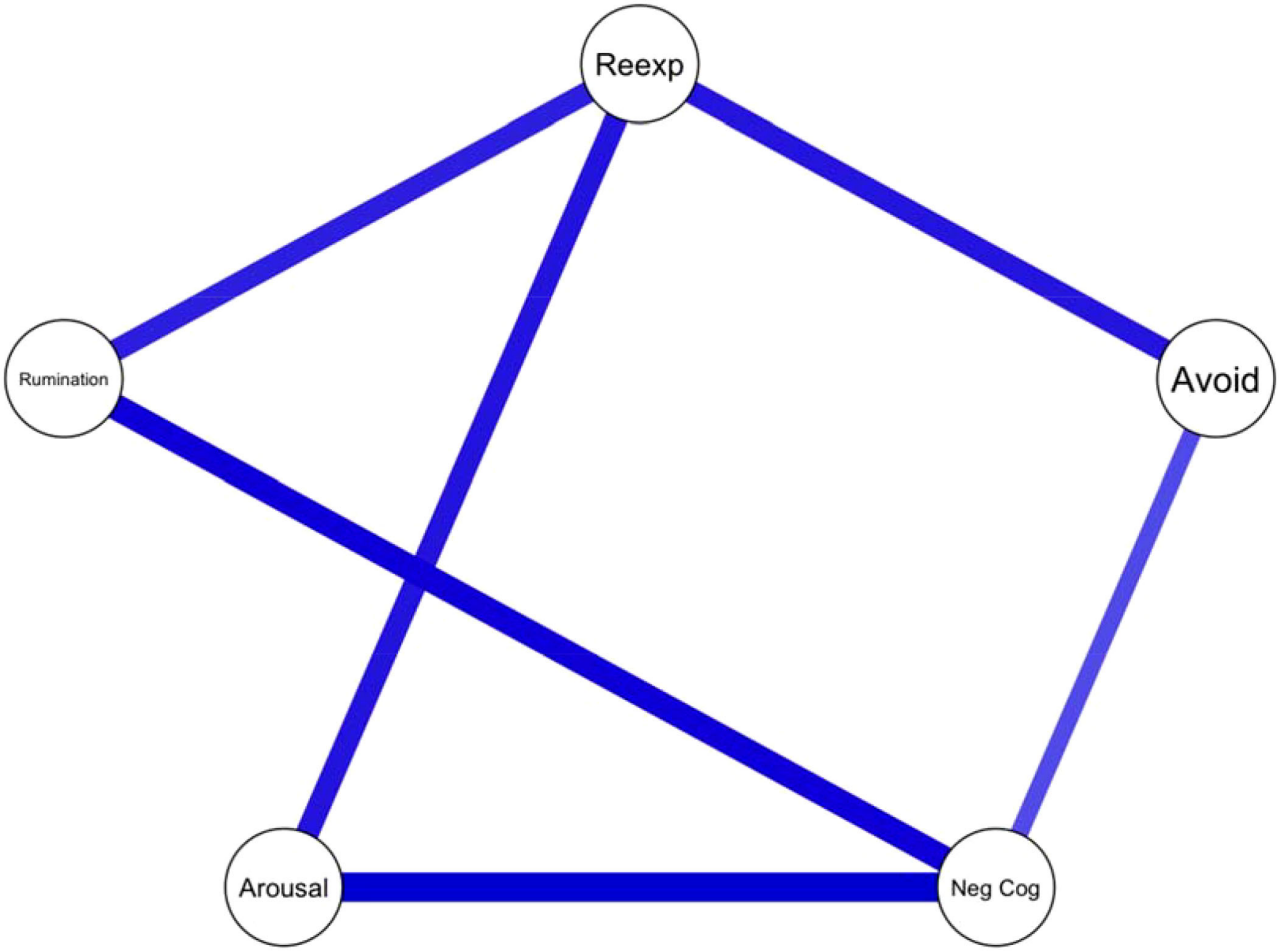
Network analysis of PTSD symptom clusters and rumination. Reexp - Reexperience, Avoid – Avoidance, Arousal – Arousal, Neg Cog – Negative Cognition.

The network between symptom clusters of PTSD symptoms and overall rumination score is depicted in **Figure 1**. All associations were positive, with rumination being related to intrusion/re-experiencing symptoms (Cluster B) and negative alterations in mood and cognition (Cluster D), but not to other clusters. In contrast, there was no direct connections with the avoidance (Cluster C) or hyperarousal clusters (Cluster E). This indicates that the relationship between PTSD and rumination is not uniform but rather dependent on specific symptom clusters.

**Figure 2:**
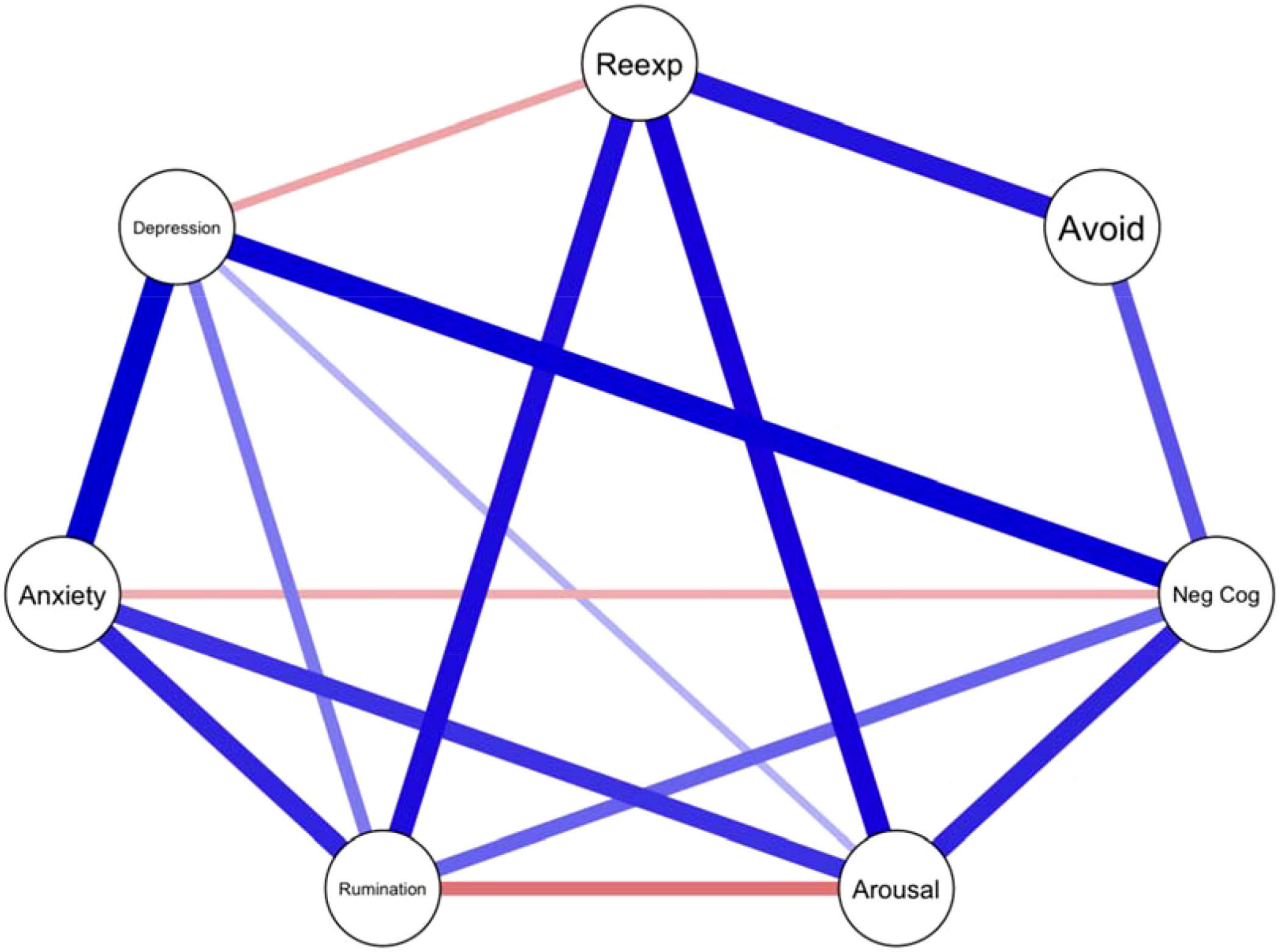
Network analysis of PTSD symptom clusters, rumination, and level of anxiety and depression. eexp - Reexperience, Avoid – Avoidance, Arousal – Arousal, Neg Cog – Negative Cognition.

The second network, shown in **Figure 2**, illustrates the associations between PTSD symptom clusters, rumination, and levels of anxiety and depression. While the edges from the first network were preserved, additional associations emerged. Notably, rumination showed a negative association with the hyperarousal cluster (Cluster E). Both depression and anxiety levels demonstrated positive associations with rumination.

**Figure 3:**
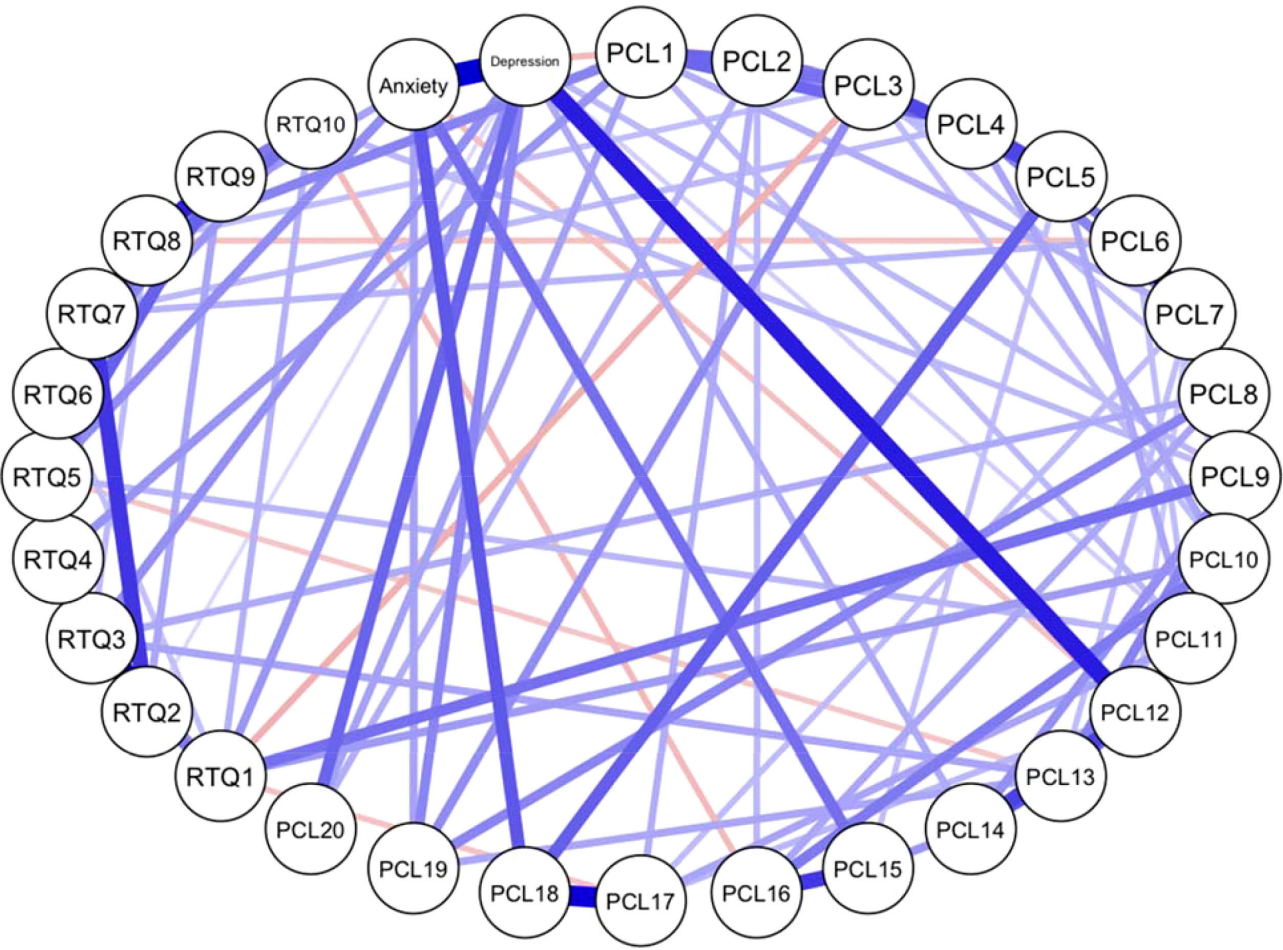
Network analysis of PTSD symptoms and rumination at the item-level, adjusted for depression and anxiety levels as additional nodes. PCL1 – Intrusive memories. PCL2 – Nightmares. PCL3 – Flashbacks. PCL4 – Emotional distress. PCL5 – Physical reactivity. PCL6 – Avoiding thoughts. PCL7 – Avoiding reminders. PCL8 – Memory gaps. PCL9 – Negative beliefs. PCL10 – Blame. PCL11 – Negative emotions. PCL12 – Anhedonia. PCL13 – Detachment. PCL14 – Emotional numbness. PCL15 – Irritability. PCL16 – Risk-taking. PCL17 – Hypervigilance. PCL18 – Startle response. PCL19 – Concentration issues. PCL20 – Sleep problems. RTQ1 – Self-criticism. RTQ2 – Intrusive thoughts. RTQ3 – Helplessness. RTQ4 – Persistent thoughts. RTQ5 – Rumination. RTQ6 – Awareness. RTQ7 – Thought resistance. RTQ8 – Preoccupation. RTQ9 – Uncontrollable thinking. RTQ10 – Wishful thinking.

**Figure 3** displays the relationship between individual PTSD symptoms and RTQ-10 items, adjusted for levels of depression and anxiety (included as nodes). This analysis revealed a dense network with predominantly positive but also some negative edges, indicating complex relationships both within and across constructs. Some relevant associations emerged between the sum score of the PHQ-9 and PCL12 (anhedonia), the sum scores between the PHQ-9 and the GAD-7, RTQ2 (intrusive thoughts) and RTQ7 (avoiding thoughts), as well as between PCL17 hypervigilance) and PCL18 (startle response) (**Supplementary Materials**). With regard to associations between PTSD and rumination, among the many connections, the following could be highlighted: A positive association was observed between PCL1 (intrusive memories) and RTQ4 (“I have thoughts or images that are difficult to forget”), highlighting potential content overlap. Conversely, a negative association was found between PCL16 (risk-taking) and RTQ10 (“I have thoughts or images about the situation and wish it would go better”), suggesting that these phenomena may be inversely related.

## 4 Discussion

Our network analysis revealed specific associations between symptoms of PTSD, depression, anxiety and rumination at both cluster and item levels, with predominantly positive but also some notable negative relationships. At the cluster level, rumination showed unique associations with re-experiencing symptoms and negative alterations in cognition and mood. Item-level analysis uncovered more complex connections, which might result from shared underlying psychological and biological mechanisms or a conceptual overlap between measurement instruments. Importantly, all findings remained after controlling for symptoms of depression and anxiety, suggesting these associations are specific to PTSD and rumination rather than attributable to shared variance with common comorbidities.

At the cluster level, rumination was associated with the re-experiencing and negative alterations in cognition and mood cluster, but not with avoidance or hyperarousal clusters, indicating a nuanced interplay between rumination and symptoms of PTSD. The relevant association between rumination and negative cognitions aligns with established literature on rumination’s role in maladaptive cognitive processes in individuals exposed to trauma (6). It is furthermore consistent with cognitive models of PTSD that conceptualize rumination as a process that reinforces negative appraisals of the trauma and its consequences (36).

Similarly, the connection between rumination and re-experiencing symptoms is consistent with previous research demonstrating associations between rumination and intrusive symptoms. For example, Birrer and Michael found that rumination was associated with more frequent intrusive images in individuals with PTSD compared to trauma-exposed controls (10). While our cross-sectional design precludes causal inferences, this pattern suggests that rumination and intrusive symptoms may be closely interconnected within the PTSD symptom network.

Our findings partially differ from a previous meta-analysis that reported an association of rumination with all PTSD symptom clusters, including avoidance and hyperarousal (9). However, those analyses did not account for the intercorrelations among PTSD symptom clusters or adjust for symptoms of depression and anxiety when estimating the strength of associations. Another meta-analysis found stronger associations between rumination and intrusion symptoms than with avoidance and hyperarousal (37), more closely aligning with our cluster-level findings. The network approach used in the current study, may provide a more precise characterization of these specific associations.

The item-level network revealed more complex relationships than were apparent at the cluster level. Despite the absence of cluster-level associations between rumination and the avoidance or arousal clusters, specific item-level connections emerged across these domains. This demonstrates that cluster-level null findings may mask critical heterogeneous item-specific associations, highlighting the value of examining relationships at multiple levels of granularity. The dense interconnections observed both within and across measurement instruments suggest that the relationships between PTSD symptoms and rumination are more nuanced than sum-score or cluster-level analyses might indicate.

Among the many associations identified, several warrant more attention due to their implications for separating rumination and intrusions. A notably association emerged between PCL1 (intrusive memories) and RTQ4 (“I have thoughts or images that are difficult to forget”). While these items showed only moderate zero-order correlations (see **Supplementary Materials**), their partial correlation in the network was substantial, indicating they are particularly closely connected after accounting for shared variance with all other variables. This pattern suggests conceptual proximity in how intrusive memories and persistent repetitive thoughts are currently measured. Although these constructs are theoretically distinct, intrusive memories typically refer to spontaneous, involuntary recall of traumatic content, whereas the RTQ item may capture more deliberate (though not necessarily controllable) perseverative thinking, their measurement appears to overlap considerably. This could be improved by integrating the deliberateness into the question asked.

Similar patterns of potentially overlapping content were observed for other item pairs in the network. For instance, RTQ1 (“thoughts or images about all your shortcomings, failings, faults, mistakes”) and PCL9 (negative beliefs) both assess negative self-referential cognitions, though one focuses on repetitive thinking about perceived failures while the other assesses broader negative beliefs. These findings imply that precise construct definition, clear theoretical distinctions, and measurement differentiation are essential when investigating overlapping trauma-related cognitive processes, particularly in the context of rumination. Future scale development should attend carefully to ensuring that items intended to measure different constructs are sufficiently distinct. They should pay particular attention to distinguish between involuntary intrusive memories, deliberate rumination about trauma, and other forms of repetitive thinking would strengthen both research and clinical practice.

A further notable negative association emerged between PCL16 (risk-taking) and RTQ10 (“I have thoughts or images about the situation and wish it would go better”). This finding is particularly important because it demonstrates the capacity of network analysis to reveal complex, non-obvious patterns that would be obscured in sum-score analyses. The pattern conceptually suggests that future-oriented, hopeful repetitive thinking (wishful thinking about improvement) may be inversely related to risk-taking behaviors. This finding challenges simplistic conceptualizations of all repetitive thinking as uniformly maladaptive in the context of trauma. Furthermore, it suggests that certain forms of repetitive thinking, particularly those with a future-oriented, hopeful quality, might function differently than backward-focused rumination about past events or their causes. This observation aligns with research distinguishing between intrusive rumination (which is typically associated with worse outcomes) and more deliberate, meaning-making forms of repetitive thinking (which can be associated with posttraumatic growth) (38).

Regarding clinical relevance, the specific associations between rumination and re-experiencing and negative cognitions clusters suggest that clinicians should routinely assess rumination in trauma-exposed individuals. When elevated rumination co-occurs with intrusive symptoms, clinicians might consider integrating rumination-focused techniques, such as functional analysis of rumination triggers or attention training, into trauma-focused protocols like CBT or EMDR. Rumination-focused cognitive-behavioral therapy (RF-CBT) may be particularly beneficial for patients showing limited response to standard trauma-focused interventions alone. To advance these findings, longitudinal studies should examine whether rumination predicts treatment response and mediates symptom improvement, while randomized trials comparing standard trauma-focused therapy with and without adjunctive rumination-focused components would clarify the added value of targeting rumination directly.

### 4.1 Limitations

There are several limitations in the current study. First, the cross-sectional design precludes inferences about temporal relationships or causality. We cannot determine whether PTSD symptoms influence rumination, or whether bidirectional relationships exist. Second, all measures were self-reported, which may introduce bias. Research demonstrates that PTSD symptoms assessed via self-report questionnaires often show inconsistencies with clinician-administered interviews, with self-report measures potentially over-identifying PTSD cases and showing limited agreement with structured clinical interviews (39). This suggests that our findings may not fully generalize to clinically diagnosed PTSD populations. Additionally, self-report measures may be influenced by response biases, memory biases, or difficulties in accurately rating symptom frequency and intensity. The exclusive reliance on self-report also prevented us from capturing clinician observations or diagnostic confirmation, which limits the clinical validity of our symptom networks. Third, the sample consisted of trauma-exposed individuals with elevated PTSD symptoms, but not all participants met full diagnostic criteria for PTSD, which limits generalizability. Fourth, this cross-sectional design precludes causal inference. Longitudinal studies are needed to examine temporal dynamics, and interventional studies to test whether targeting rumination affects PTSD symptom trajectories. Finally, the RTQ-10 is a general measure of repetitive negative thinking rather than a PTSD-specific rumination assessment. While this transdiagnostic measure allowed us to capture repetitive thinking processes without being limited to disorder-specific content, it does not distinguish between rumination subtypes that may have differential relationships with PTSD symptoms. For instance, intrusive rumination (involuntary, automatic negative thoughts about the trauma) and deliberate rumination (effortful attempts at meaning-making) may show different patterns of association with specific PTSD symptoms (38), as might brooding versus reflection (12). Our measure did not capture these distinctions, which limits the theoretical sensitivity of our findings.

## 5 Conclusion

This multi-level network analysis revealed that rumination shows selective associations with specific PTSD symptom clusters, particularly re-experiencing and negative cognitions, rather than being uniformly associated with all aspects of PTSD. These associations remained after controlling for depression and anxiety symptoms, suggesting they reflect specific relationships rather than shared variance with common comorbidities. Item-level analysis uncovered additional complexity, including cross-cluster connections not apparent at the cluster level and relevant associations between items that may reflect both substantive psychological relationships and conceptual overlap in measurement. The identification of a negative association between risk-taking and wishful thinking further demonstrates how network analysis can reveal nuanced patterns missed by aggregate approaches. These findings highlight the importance of precise construct definition and measurement differentiation when investigating trauma-related cognitive processes and establish groundwork for future longitudinal and treatment research examining the clinical implications of these specific PTSD-rumination connections.

## Data Availability

The data supporting the results of this manuscript are available from the senior author, IH-R, upon request and subject to approval by all parties involved in the study, including the IRB at Yale University.

## References

[1] Davis LL Schein J Cloutier M Gagnon-Sanschagrin P Maitland J Urganus A . The economic burden of posttraumatic stress disorder in the United States from a societal perspective. J Clin Psychiatry. (2022) 83:21m14116. doi: 10.4088/JCP.21m14116, PMID: 35485933

[2] American Psychiatric Association . Diagnostic and Statistical Manual of Mental Disorders, Fifth Edition, Text Revision (DSM-5-TR). 5th ed. Arlington, VA: American Psychiatric Association Publishing (2022). p. 1120.

[3] Brady KT Killeen TK Brewerton T Lucerini S . Comorbidity of psychiatric disorders and posttraumatic stress disorder. J Clin Psychiatry. (2000) 61:22–32. 10795606

[4] Rickerby N Krug I Fuller-Tyszkiewicz M Forte E Davenport R Chayadi E . Rumination across depression, anxiety, and eating disorders in adults: A meta-analytic review. Clin Psychol Sci Pract. (2024) 31:251–68. doi: 10.1037/cps0000110, PMID: 41770175

[5] Ehring T Watkins ER . Repetitive negative thinking as a transdiagnostic process. Int J Cognit Ther. (2008) 1:192–205. doi: 10.1521/ijct.2008.1.3.192, PMID: 39183330

[6] Moulds ML Bisby MA Wild J Bryant RA . Rumination in posttraumatic stress disorder: A systematic review. Clin Psychol Rev. (2020) 82:101910. doi: 10.1016/j.cpr.2020.101910, PMID: 32971312

[7] Michael T Halligan SL Clark DM Ehlers A . Rumination in posttraumatic stress disorder. Depress Anxiety. (2007) 24:307–17. doi: 10.1002/da.20228, PMID: 17041914

[8] Ehring T Frank S Ehlers A . The role of rumination and reduced concreteness in the maintenance of posttraumatic stress disorder and depression following trauma. Cognit Ther Res. (2008) 32:488–506. doi: 10.1007/s10608-006-9089-7, PMID: 20694036 PMC2908437

[9] Miethe S Wigger J Wartemann A Fuchs FO Trautmann S . Posttraumatic stress symptoms and its association with rumination, thought suppression and experiential avoidance: a systematic review and meta-analysis. J Psychopathol Behav Assess. (2023) 45:480–95. doi: 10.1007/s10862-023-10022-2, PMID: 41868966

[10] Birrer E Michael T . Rumination in PTSD as well as in traumatized and non-traumatized depressed patients: a cross-sectional clinical study. Behav Cognit Psychother. (2011) 39:381–97. doi: 10.1017/S1352465811000087, PMID: 21457604

[11] Nichter B Norman S Haller M Pietrzak RH . Psychological burden of PTSD, depression, and their comorbidity in the U.S. veteran population: Suicidality, functioning, and service utilization. J Affect Disord. (2019) 256:633–40. doi: 10.1016/j.jad.2019.06.072, PMID: 31299445

[12] Roley ME Claycomb MA Contractor AA Dranger P Armour C Elhai JD . The relationship between rumination, PTSD, and depression symptoms. J Affect Disord. (2015) 180:116–21. doi: 10.1016/j.jad.2015.04.006, PMID: 25898331

[13] Ródenas-Perea G Velasco-Barbancho E Perona-Garcelán S Rodríguez-Testal JF Senín-Calderón C Crespo-Facorro B . Childhood and adolescent trauma and dissociation: The mediating role of rumination, intrusive thoughts and negative affect. Scand J Psychol. (2023) 64:142–9. doi: 10.1111/sjop.12879, PMID: 36240326

[14] Kim JS Jin MJ Jung W Hahn SW Lee SH . Rumination as a mediator between childhood trauma and adulthood depression/anxiety in non-clinical participants. Front Psychol. (2017) 8:1597/full. doi: 10.3389/fpsyg.2017.01597/full 28993746 PMC5622198

[15] Arditte Hall KA Davison EH Galovski TE Vasterling JJ Pineles SL . Associations between trauma-related rumination and symptoms of posttraumatic stress and depression in treatment-seeking female veterans. J Trauma Stress. (2019) 32:260–8. doi: 10.1002/jts.22385, PMID: 31009555

[16] Mendoza NB Mordeno IG Nalipay MJN . The transdiagnostic role of rumination in the comorbidity of PTSD and depression. J Loss Trauma. (2022) 27:731–45. doi: 10.1080/15325024.2021.2018197, PMID: 41858497

[17] Borsboom D . A network theory of mental disorders. World Psychiatry. (2017) 16:5–13. doi: 10.1002/wps.20375, PMID: 28127906 PMC5269502

[18] Borsboom D Deserno MK Rhemtulla M Epskamp S Fried EI McNally RJ . Network analysis of multivariate data in psychological science. Nat Rev Methods Primer. (2021) 1:1–1. doi: 10.1038/s43586-021-00055-w, PMID: 41862587

[19] Fried EI van Borkulo CD Cramer AOJ Boschloo L Schoevers RA Borsboom D . Mental disorders as networks of problems: a review of recent insights. Soc Psychiatry Psychiatr Epidemiol. (2017) 52:1–10. doi: 10.1007/s00127-016-1319-z, PMID: 27921134 PMC5226976

[20] McNally RJ . Can network analysis transform psychopathology? Behav Res Ther. (2016) 86:95–104. 27424882 10.1016/j.brat.2016.06.006

[21] Fried EI Eidhof MB Palic S Costantini G Huisman-van Dijk HM Bockting CLH . Replicability and generalizability of posttraumatic stress disorder (PTSD) networks: A cross-cultural multisite study of PTSD symptoms in four trauma patient samples. Clin Psychol Sci. (2018) 6:335–51. doi: 10.1177/2167702617745092, PMID: 29881651 PMC5974702

[22] Spiller TR Schick M Schnyder U Bryant RA Nickerson A Morina N . Symptoms of posttraumatic stress disorder in a clinical sample of refugees: a network analysis. Eur J Psychotraumatology. (2017) 8:1318032. doi: 10.1080/20008198.2017.1318032, PMID: 29038688 PMC5639426

[23] Greene T Gelkopf M Epskamp S Fried E . Dynamic networks of PTSD symptoms during conflict. Psychol Med. (2018) 48:2409–17. doi: 10.1017/S0033291718000351, PMID: 29486811

[24] Duek O Spiller TR Pietrzak RH Fried EI Harpaz-Rotem I . Network analysis of PTSD and depressive symptoms in 158,139 treatment-seeking veterans with PTSD. Depress Anxiety. (2020) 38:554–62. 10.1002/da.23112 33190348

[25] Price M Legrand AC Brier ZMF Hébert-Dufresne L . The symptoms at the center: Examining the comorbidity of posttraumatic stress disorder, generalized anxiety disorder, and depression with network analysis. J Psychiatr Res. (2019) 109:52–8. doi: 10.1016/j.jpsychires.2018.11.016, PMID: 30502492 PMC6420212

[26] Collins AC Lass ANS Jordan DG Winer ES . Examining rumination, devaluation of positivity, and depressive symptoms via community-based network analysis. J Clin Psychol. (2021) 77:2228–44. doi: 10.1002/jclp.23158, PMID: 33960420

[27] Bernstein EE Heeren A McNally RJ . Reexamining trait rumination as a system of repetitive negative thoughts: A network analysis. J Behav Ther Exp Psychiatry. (2019) 63:21–7. doi: 10.1016/j.jbtep.2018.12.005, PMID: 30590225

[28] Palan S Schitter C . Prolific.ac—A subject pool for online experiments. J Behav Exp Finance. (2018) 17:22–7. doi: 10.1016/j.jbef.2017.12.004, PMID: 41869561

[29] Blevins CA Weathers FW Davis MT Witte TK Domino JL . The posttraumatic stress disorder checklist for *DSM-5* (PCL-5): development and initial psychometric evaluation. J Trauma Stress. (2015) 28:489–98. doi: 10.1002/jts.22059, PMID: 26606250

[30] McEvoy PM Hyett MP Ehring T Johnson SL Samtani S Anderson R . Transdiagnostic assessment of repetitive negative thinking and responses to positive affect: Structure and predictive utility for depression, anxiety, and mania symptoms. J Affect Disord. (2018) 232:375–84. doi: 10.1016/j.jad.2018.02.072, PMID: 29510356

[31] Mahoney AEJ McEvoy PM Moulds ML . Psychometric properties of the Repetitive Thinking Questionnaire in a clinical sample. J Anxiety Disord. (2012) 26:359–67. doi: 10.1016/j.janxdis.2011.12.003, PMID: 22204788

[32] Spitzer RL Kroenke K Williams JBW Löwe B . A brief measure for assessing generalized anxiety disorder: the GAD-7. Arch Intern Med. (2006) 166:1092. doi: 10.1001/archinte.166.10.1092, PMID: 16717171

[33] Kroenke K Spitzer RL Williams JBW . The PHQ-9: Validity of a brief depression severity measure. J Gen Intern Med. (2001) 16:606–13. doi: 10.1046/j.1525-1497.2001.016009606.x, PMID: 11556941 PMC1495268

[34] Epskamp S Borsboom D Fried EI . Estimating psychological networks and their accuracy: A tutorial paper. Behav Res Methods. (2018) 50:195–212. doi: 10.3758/s13428-017-0862-1, PMID: 28342071 PMC5809547

[35] Foygel R Drton M . Extended bayesian information criteria for gaussian graphical models. arXiv. (2010). Available online at: http://arxiv.org/abs/1011.6640 (Accessed November 14, 2025).

[36] Ehlers A Clark DM . A cognitive model of posttraumatic stress disorder. Behav Res Ther. (2000) 38:319–45. doi: 10.1016/S0005-7967(99)00123-0, PMID: 10761279

[37] Szabo YZ Warnecke AJ Newton TL Valentine JC . Rumination and posttraumatic stress symptoms in trauma-exposed adults: a systematic review and meta-analysis. Anxiety Stress Coping. (2017) 30:396–414. doi: 10.1080/10615806.2017.1313835, PMID: 28398085

[38] Zhou X Wu X . The relationship between rumination, posttraumatic stress disorder, and posttraumatic growth among Chinese adolescents after earthquake: A longitudinal study. J Affect Disord. (2016) 193:242–8. doi: 10.1016/j.jad.2015.12.076, PMID: 26773915

[39] Kramer LB Whiteman SE Petri JM Spitzer EG Weathers FW . Self-rated versus clinician-rated assessment of posttraumatic stress disorder: an evaluation of discrepancies between the PTSD checklist for *DSM-5* and the clinician-administered PTSD scale for DSM-5. Assessment. (2023) 30:1590–605. doi: 10.1177/10731911221113571, PMID: 35915927

