# Supplementary material for: Relationship between rumination and symptoms of posttraumatic stress disorder – a cross-sectional network analysis
Source: Front Psychiatry. 2026 Mar 24;17:1786768. doi: 10.3389/fpsyt.2026.1786768 (PMC13054884; doi:10.3389/fpsyt.2026.1786768)
Supplement: Supplementary file 1 [file Supplementaryfile1.docx]

**Supplementary Materials No. 1 for**

**Relationship between Rumination and Symptoms of Posttraumatic Stress Disorder – a cross-sectional network analysis**

Olena Zhabenko; Ziv Ben-Zion; Paul Raffelhüschen; Achim Burrer; Erich Seifritz; Stefan Jost, Kathrin Preller; Or Duek; Robert H. Pietryak; Jutta Joormann; Tobias R. Spiller; Ilan Harpaz-Rotem

**Summary:**

These supplementary material No. 1 contains additional depictions of the PTSD networks and the results of their stability and reliability analyses.

**Supplementary Table 1.** Zero order correlation matrix of all items included in the network analysis

|  | PCL1 | PCL2 | PCL3 | PCL4 | PCL5 | PCL6 | PCL7 | PCL8 | PCL9 | PCL10 | PCL11 | PCL12 | PCL13 | PCL14 | PCL15 | PCL16 | PCL17 | PCL18 | PCL19 | PCL20 |
| --- | --- | --- | --- | --- | --- | --- | --- | --- | --- | --- | --- | --- | --- | --- | --- | --- | --- | --- | --- | --- |
| PCL1 | 1,000 | 0,421 | 0,466 | 0,498 | 0,428 | 0,350 | 0,417 | 0,163 | 0,288 | 0,365 | 0,402 | 0,280 | 0,263 | 0,256 | 0,174 | 0,117 | 0,302 | 0,311 | 0,278 | 0,322 |
| PCL2 | 0,421 | 1,000 | 0,444 | 0,293 | 0,372 | 0,219 | 0,265 | 0,164 | 0,177 | 0,236 | 0,260 | 0,182 | 0,133 | 0,171 | 0,122 | 0,177 | 0,268 | 0,280 | 0,193 | 0,252 |
| PCL3 | 0,466 | 0,444 | 1,000 | 0,420 | 0,479 | 0,260 | 0,343 | 0,241 | 0,187 | 0,326 | 0,338 | 0,204 | 0,225 | 0,240 | 0,228 | 0,209 | 0,266 | 0,306 | 0,330 | 0,182 |
| PCL4 | 0,498 | 0,293 | 0,420 | 1,000 | 0,491 | 0,466 | 0,499 | 0,207 | 0,377 | 0,405 | 0,415 | 0,278 | 0,289 | 0,309 | 0,210 | 0,101 | 0,275 | 0,283 | 0,298 | 0,208 |
| PCL5 | 0,428 | 0,372 | 0,479 | 0,491 | 1,000 | 0,303 | 0,394 | 0,210 | 0,257 | 0,305 | 0,399 | 0,227 | 0,248 | 0,254 | 0,266 | 0,148 | 0,365 | 0,418 | 0,312 | 0,233 |
| PCL6 | 0,350 | 0,219 | 0,260 | 0,466 | 0,303 | 1,000 | 0,563 | 0,274 | 0,282 | 0,342 | 0,345 | 0,236 | 0,248 | 0,212 | 0,176 | 0,091 | 0,234 | 0,180 | 0,234 | 0,202 |
| PCL7 | 0,417 | 0,265 | 0,343 | 0,499 | 0,394 | 0,563 | 1,000 | 0,225 | 0,364 | 0,372 | 0,327 | 0,358 | 0,368 | 0,316 | 0,179 | 0,126 | 0,307 | 0,258 | 0,285 | 0,241 |
| PCL8 | 0,163 | 0,164 | 0,241 | 0,207 | 0,210 | 0,274 | 0,225 | 1,000 | 0,181 | 0,225 | 0,243 | 0,185 | 0,201 | 0,185 | 0,157 | 0,204 | 0,155 | 0,244 | 0,313 | 0,097 |
| PCL9 | 0,288 | 0,177 | 0,187 | 0,377 | 0,257 | 0,282 | 0,364 | 0,181 | 1,000 | 0,417 | 0,480 | 0,422 | 0,483 | 0,474 | 0,287 | 0,159 | 0,271 | 0,245 | 0,359 | 0,220 |
| PCL10 | 0,365 | 0,236 | 0,326 | 0,405 | 0,305 | 0,342 | 0,372 | 0,225 | 0,417 | 1,000 | 0,484 | 0,350 | 0,367 | 0,334 | 0,251 | 0,257 | 0,235 | 0,229 | 0,298 | 0,257 |
| PCL11 | 0,402 | 0,260 | 0,338 | 0,415 | 0,399 | 0,345 | 0,327 | 0,243 | 0,480 | 0,484 | 1,000 | 0,380 | 0,368 | 0,388 | 0,262 | 0,164 | 0,338 | 0,324 | 0,344 | 0,244 |
| PCL12 | 0,280 | 0,182 | 0,204 | 0,278 | 0,227 | 0,236 | 0,358 | 0,185 | 0,422 | 0,350 | 0,380 | 1,000 | 0,538 | 0,579 | 0,326 | 0,217 | 0,296 | 0,268 | 0,419 | 0,306 |
| PCL13 | 0,263 | 0,133 | 0,225 | 0,289 | 0,248 | 0,248 | 0,368 | 0,201 | 0,483 | 0,367 | 0,368 | 0,538 | 1,000 | 0,545 | 0,269 | 0,189 | 0,285 | 0,268 | 0,386 | 0,306 |
| PCL14 | 0,256 | 0,171 | 0,240 | 0,309 | 0,254 | 0,212 | 0,316 | 0,185 | 0,474 | 0,334 | 0,388 | 0,579 | 0,545 | 1,000 | 0,325 | 0,225 | 0,280 | 0,266 | 0,369 | 0,279 |
| PCL15 | 0,174 | 0,122 | 0,228 | 0,210 | 0,266 | 0,176 | 0,179 | 0,157 | 0,287 | 0,251 | 0,262 | 0,326 | 0,269 | 0,325 | 1,000 | 0,354 | 0,207 | 0,281 | 0,309 | 0,188 |
| PCL16 | 0,117 | 0,177 | 0,209 | 0,101 | 0,148 | 0,091 | 0,126 | 0,204 | 0,159 | 0,257 | 0,164 | 0,217 | 0,189 | 0,225 | 0,354 | 1,000 | 0,142 | 0,147 | 0,213 | 0,138 |
| PCL17 | 0,302 | 0,268 | 0,266 | 0,275 | 0,365 | 0,234 | 0,307 | 0,155 | 0,271 | 0,235 | 0,338 | 0,296 | 0,285 | 0,280 | 0,207 | 0,142 | 1,000 | 0,491 | 0,238 | 0,215 |
| PCL18 | 0,311 | 0,280 | 0,306 | 0,283 | 0,418 | 0,180 | 0,258 | 0,244 | 0,245 | 0,229 | 0,324 | 0,268 | 0,268 | 0,266 | 0,281 | 0,147 | 0,491 | 1,000 | 0,320 | 0,238 |
| PCL19 | 0,278 | 0,193 | 0,330 | 0,298 | 0,312 | 0,234 | 0,285 | 0,313 | 0,359 | 0,298 | 0,344 | 0,419 | 0,386 | 0,369 | 0,309 | 0,213 | 0,238 | 0,320 | 1,000 | 0,273 |
| PCL20 | 0,322 | 0,252 | 0,182 | 0,208 | 0,233 | 0,202 | 0,241 | 0,097 | 0,220 | 0,257 | 0,244 | 0,306 | 0,306 | 0,279 | 0,188 | 0,138 | 0,215 | 0,238 | 0,273 | 1,000 |
| RTQ1 | 0,278 | 0,146 | 0,173 | 0,334 | 0,227 | 0,212 | 0,258 | 0,150 | 0,512 | 0,386 | 0,394 | 0,384 | 0,344 | 0,352 | 0,227 | 0,102 | 0,142 | 0,182 | 0,305 | 0,270 |
| RTQ2 | 0,446 | 0,194 | 0,242 | 0,365 | 0,317 | 0,312 | 0,342 | 0,116 | 0,372 | 0,339 | 0,399 | 0,285 | 0,343 | 0,297 | 0,184 | 0,026 | 0,214 | 0,245 | 0,290 | 0,259 |
| RTQ3 | 0,292 | 0,240 | 0,310 | 0,332 | 0,320 | 0,206 | 0,316 | 0,282 | 0,428 | 0,339 | 0,391 | 0,398 | 0,392 | 0,350 | 0,259 | 0,199 | 0,231 | 0,318 | 0,396 | 0,236 |
| RTQ4 | 0,472 | 0,207 | 0,297 | 0,381 | 0,312 | 0,314 | 0,378 | 0,157 | 0,363 | 0,345 | 0,398 | 0,288 | 0,301 | 0,302 | 0,154 | 0,074 | 0,165 | 0,227 | 0,274 | 0,253 |
| RTQ5 | 0,417 | 0,218 | 0,279 | 0,401 | 0,353 | 0,229 | 0,276 | 0,178 | 0,421 | 0,321 | 0,450 | 0,309 | 0,274 | 0,323 | 0,253 | 0,148 | 0,207 | 0,277 | 0,375 | 0,236 |
| RTQ6 | 0,398 | 0,192 | 0,284 | 0,387 | 0,314 | 0,247 | 0,337 | 0,096 | 0,363 | 0,344 | 0,399 | 0,265 | 0,277 | 0,298 | 0,193 | 0,039 | 0,223 | 0,233 | 0,277 | 0,236 |
| RTQ7 | 0,449 | 0,232 | 0,272 | 0,438 | 0,375 | 0,363 | 0,396 | 0,138 | 0,370 | 0,334 | 0,403 | 0,275 | 0,304 | 0,302 | 0,194 | 0,065 | 0,237 | 0,274 | 0,261 | 0,286 |
| RTQ8 | 0,480 | 0,299 | 0,389 | 0,371 | 0,368 | 0,217 | 0,289 | 0,184 | 0,405 | 0,335 | 0,435 | 0,333 | 0,325 | 0,331 | 0,226 | 0,120 | 0,226 | 0,266 | 0,353 | 0,253 |
| RTQ9 | 0,423 | 0,221 | 0,294 | 0,395 | 0,332 | 0,269 | 0,335 | 0,129 | 0,406 | 0,327 | 0,359 | 0,295 | 0,301 | 0,296 | 0,205 | 0,072 | 0,211 | 0,244 | 0,345 | 0,270 |
| RTQ10 | 0,368 | 0,178 | 0,276 | 0,383 | 0,307 | 0,241 | 0,315 | 0,088 | 0,422 | 0,334 | 0,365 | 0,324 | 0,275 | 0,300 | 0,159 | 0,019 | 0,199 | 0,243 | 0,332 | 0,189 |
| RHQ1 | 0,181 | 0,119 | 0,144 | 0,227 | 0,183 | 0,227 | 0,253 | 0,165 | 0,429 | 0,297 | 0,342 | 0,699 | 0,458 | 0,552 | 0,303 | 0,170 | 0,229 | 0,229 | 0,393 | 0,251 |
| PHQ2 | 0,233 | 0,163 | 0,193 | 0,308 | 0,233 | 0,246 | 0,263 | 0,137 | 0,500 | 0,340 | 0,359 | 0,558 | 0,416 | 0,529 | 0,285 | 0,097 | 0,206 | 0,233 | 0,340 | 0,274 |
| PHQ3 | 0,243 | 0,145 | 0,158 | 0,176 | 0,169 | 0,177 | 0,183 | 0,081 | 0,274 | 0,219 | 0,251 | 0,327 | 0,289 | 0,291 | 0,204 | 0,100 | 0,196 | 0,194 | 0,268 | 0,670 |
| PHQ4 | 0,186 | 0,078 | 0,107 | 0,220 | 0,187 | 0,239 | 0,220 | 0,161 | 0,348 | 0,233 | 0,258 | 0,420 | 0,321 | 0,348 | 0,288 | 0,080 | 0,176 | 0,234 | 0,371 | 0,334 |
| PHQ5 | 0,203 | 0,140 | 0,211 | 0,158 | 0,207 | 0,143 | 0,177 | 0,145 | 0,250 | 0,220 | 0,296 | 0,356 | 0,309 | 0,277 | 0,271 | 0,123 | 0,162 | 0,194 | 0,286 | 0,252 |
| PHQ6 | 0,246 | 0,167 | 0,186 | 0,291 | 0,216 | 0,244 | 0,273 | 0,145 | 0,578 | 0,387 | 0,426 | 0,429 | 0,424 | 0,406 | 0,221 | 0,105 | 0,205 | 0,236 | 0,295 | 0,213 |
| PHQ7 | 0,240 | 0,155 | 0,247 | 0,250 | 0,247 | 0,192 | 0,254 | 0,257 | 0,330 | 0,249 | 0,317 | 0,397 | 0,337 | 0,372 | 0,279 | 0,203 | 0,249 | 0,303 | 0,640 | 0,261 |
| PHQ8 | 0,254 | 0,232 | 0,298 | 0,184 | 0,251 | 0,168 | 0,204 | 0,242 | 0,207 | 0,227 | 0,265 | 0,305 | 0,210 | 0,266 | 0,286 | 0,300 | 0,208 | 0,259 | 0,342 | 0,142 |
| PHQ9 | 0,197 | 0,226 | 0,200 | 0,201 | 0,220 | 0,141 | 0,179 | 0,176 | 0,408 | 0,312 | 0,337 | 0,341 | 0,325 | 0,364 | 0,266 | 0,229 | 0,129 | 0,205 | 0,241 | 0,149 |
| GAD1 | 0,258 | 0,152 | 0,184 | 0,276 | 0,287 | 0,199 | 0,228 | 0,107 | 0,359 | 0,257 | 0,356 | 0,333 | 0,278 | 0,334 | 0,271 | 0,048 | 0,294 | 0,399 | 0,372 | 0,227 |
| GAD2 | 0,365 | 0,196 | 0,244 | 0,344 | 0,311 | 0,226 | 0,259 | 0,183 | 0,411 | 0,302 | 0,354 | 0,358 | 0,301 | 0,349 | 0,330 | 0,115 | 0,286 | 0,391 | 0,406 | 0,318 |
| GAD3 | 0,348 | 0,209 | 0,236 | 0,345 | 0,324 | 0,289 | 0,297 | 0,175 | 0,393 | 0,321 | 0,351 | 0,349 | 0,316 | 0,325 | 0,360 | 0,114 | 0,287 | 0,346 | 0,421 | 0,290 |
| GAD4 | 0,300 | 0,181 | 0,191 | 0,296 | 0,281 | 0,205 | 0,190 | 0,183 | 0,339 | 0,262 | 0,309 | 0,368 | 0,313 | 0,387 | 0,281 | 0,125 | 0,243 | 0,305 | 0,383 | 0,414 |
| GAD5 | 0,269 | 0,185 | 0,260 | 0,232 | 0,293 | 0,161 | 0,213 | 0,223 | 0,255 | 0,248 | 0,266 | 0,291 | 0,271 | 0,324 | 0,319 | 0,272 | 0,284 | 0,333 | 0,364 | 0,234 |
| GAD6 | 0,173 | 0,056 | 0,128 | 0,228 | 0,208 | 0,182 | 0,169 | 0,113 | 0,289 | 0,228 | 0,270 | 0,282 | 0,212 | 0,305 | 0,542 | 0,175 | 0,192 | 0,289 | 0,281 | 0,164 |
| GAD7 | 0,335 | 0,233 | 0,329 | 0,372 | 0,375 | 0,225 | 0,269 | 0,147 | 0,387 | 0,302 | 0,442 | 0,346 | 0,245 | 0,315 | 0,241 | 0,112 | 0,366 | 0,446 | 0,363 | 0,178 |

**ff. Supplementary Table 1.** Zero order correlation matrix of all items included in the network analysis

|  | RTQ1 | RTQ2 | RTQ3 | RTQ4 | RTQ5 | RTQ6 | RTQ7 | RTQ8 | RTQ9 | RTQ10 | PHQ1 | PHQ2 | PHQ3 | PHQ4 | PHQ5 | PHQ6 | PHQ7 | PHQ8 | PHQ9 | GAD1 | GAD2 | GAD3 | GAD4 | GAD5 | GAD6 | GAD7 |
| --- | --- | --- | --- | --- | --- | --- | --- | --- | --- | --- | --- | --- | --- | --- | --- | --- | --- | --- | --- | --- | --- | --- | --- | --- | --- | --- |
| PCL1 | 0,278 | 0,446 | 0,292 | 0,472 | 0,417 | 0,398 | 0,449 | 0,480 | 0,423 | 0,368 | 0,181 | 0,233 | 0,243 | 0,186 | 0,203 | 0,246 | 0,240 | 0,254 | 0,197 | 0,258 | 0,365 | 0,348 | 0,300 | 0,269 | 0,173 | 0,335 |
| PCL2 | 0,146 | 0,194 | 0,240 | 0,207 | 0,218 | 0,192 | 0,232 | 0,299 | 0,221 | 0,178 | 0,119 | 0,163 | 0,145 | 0,078 | 0,140 | 0,167 | 0,155 | 0,232 | 0,226 | 0,152 | 0,196 | 0,209 | 0,181 | 0,185 | 0,056 | 0,233 |
| PCL3 | 0,173 | 0,242 | 0,310 | 0,297 | 0,279 | 0,284 | 0,272 | 0,389 | 0,294 | 0,276 | 0,144 | 0,193 | 0,158 | 0,107 | 0,211 | 0,186 | 0,247 | 0,298 | 0,200 | 0,184 | 0,244 | 0,236 | 0,191 | 0,260 | 0,128 | 0,329 |
| PCL4 | 0,334 | 0,365 | 0,332 | 0,381 | 0,401 | 0,387 | 0,438 | 0,371 | 0,395 | 0,383 | 0,227 | 0,308 | 0,176 | 0,220 | 0,158 | 0,291 | 0,250 | 0,184 | 0,201 | 0,276 | 0,344 | 0,345 | 0,296 | 0,232 | 0,228 | 0,372 |
| PCL5 | 0,227 | 0,317 | 0,320 | 0,312 | 0,353 | 0,314 | 0,375 | 0,368 | 0,332 | 0,307 | 0,183 | 0,233 | 0,169 | 0,187 | 0,207 | 0,216 | 0,247 | 0,251 | 0,220 | 0,287 | 0,311 | 0,324 | 0,281 | 0,293 | 0,208 | 0,375 |
| PCL6 | 0,212 | 0,312 | 0,206 | 0,314 | 0,229 | 0,247 | 0,363 | 0,217 | 0,269 | 0,241 | 0,227 | 0,246 | 0,177 | 0,239 | 0,143 | 0,244 | 0,192 | 0,168 | 0,141 | 0,199 | 0,226 | 0,289 | 0,205 | 0,161 | 0,182 | 0,225 |
| PCL7 | 0,258 | 0,342 | 0,316 | 0,378 | 0,276 | 0,337 | 0,396 | 0,289 | 0,335 | 0,315 | 0,253 | 0,263 | 0,183 | 0,220 | 0,177 | 0,273 | 0,254 | 0,204 | 0,179 | 0,228 | 0,259 | 0,297 | 0,190 | 0,213 | 0,169 | 0,269 |
| PCL8 | 0,150 | 0,116 | 0,282 | 0,157 | 0,178 | 0,096 | 0,138 | 0,184 | 0,129 | 0,088 | 0,165 | 0,137 | 0,081 | 0,161 | 0,145 | 0,145 | 0,257 | 0,242 | 0,176 | 0,107 | 0,183 | 0,175 | 0,183 | 0,223 | 0,113 | 0,147 |
| PCL9 | 0,512 | 0,372 | 0,428 | 0,363 | 0,421 | 0,363 | 0,370 | 0,405 | 0,406 | 0,422 | 0,429 | 0,500 | 0,274 | 0,348 | 0,250 | 0,578 | 0,330 | 0,207 | 0,408 | 0,359 | 0,411 | 0,393 | 0,339 | 0,255 | 0,289 | 0,387 |
| PCL10 | 0,386 | 0,339 | 0,339 | 0,345 | 0,321 | 0,344 | 0,334 | 0,335 | 0,327 | 0,334 | 0,297 | 0,340 | 0,219 | 0,233 | 0,220 | 0,387 | 0,249 | 0,227 | 0,312 | 0,257 | 0,302 | 0,321 | 0,262 | 0,248 | 0,228 | 0,302 |
| PCL11 | 0,394 | 0,399 | 0,391 | 0,398 | 0,450 | 0,399 | 0,403 | 0,435 | 0,359 | 0,365 | 0,342 | 0,359 | 0,251 | 0,258 | 0,296 | 0,426 | 0,317 | 0,265 | 0,337 | 0,356 | 0,354 | 0,351 | 0,309 | 0,266 | 0,270 | 0,442 |
| PCL12 | 0,384 | 0,285 | 0,398 | 0,288 | 0,309 | 0,265 | 0,275 | 0,333 | 0,295 | 0,324 | 0,699 | 0,558 | 0,327 | 0,420 | 0,356 | 0,429 | 0,397 | 0,305 | 0,341 | 0,333 | 0,358 | 0,349 | 0,368 | 0,291 | 0,282 | 0,346 |
| PCL13 | 0,344 | 0,343 | 0,392 | 0,301 | 0,274 | 0,277 | 0,304 | 0,325 | 0,301 | 0,275 | 0,458 | 0,416 | 0,289 | 0,321 | 0,309 | 0,424 | 0,337 | 0,210 | 0,325 | 0,278 | 0,301 | 0,316 | 0,313 | 0,271 | 0,212 | 0,245 |
| PCL14 | 0,352 | 0,297 | 0,350 | 0,302 | 0,323 | 0,298 | 0,302 | 0,331 | 0,296 | 0,300 | 0,552 | 0,529 | 0,291 | 0,348 | 0,277 | 0,406 | 0,372 | 0,266 | 0,364 | 0,334 | 0,349 | 0,325 | 0,387 | 0,324 | 0,305 | 0,315 |
| PCL15 | 0,227 | 0,184 | 0,259 | 0,154 | 0,253 | 0,193 | 0,194 | 0,226 | 0,205 | 0,159 | 0,303 | 0,285 | 0,204 | 0,288 | 0,271 | 0,221 | 0,279 | 0,286 | 0,266 | 0,271 | 0,330 | 0,360 | 0,281 | 0,319 | 0,542 | 0,241 |
| PCL16 | 0,102 | 0,026 | 0,199 | 0,074 | 0,148 | 0,039 | 0,065 | 0,120 | 0,072 | 0,019 | 0,170 | 0,097 | 0,100 | 0,080 | 0,123 | 0,105 | 0,203 | 0,300 | 0,229 | 0,048 | 0,115 | 0,114 | 0,125 | 0,272 | 0,175 | 0,112 |
| PCL17 | 0,142 | 0,214 | 0,231 | 0,165 | 0,207 | 0,223 | 0,237 | 0,226 | 0,211 | 0,199 | 0,229 | 0,206 | 0,196 | 0,176 | 0,162 | 0,205 | 0,249 | 0,208 | 0,129 | 0,294 | 0,286 | 0,287 | 0,243 | 0,284 | 0,192 | 0,366 |
| PCL18 | 0,182 | 0,245 | 0,318 | 0,227 | 0,277 | 0,233 | 0,274 | 0,266 | 0,244 | 0,243 | 0,229 | 0,233 | 0,194 | 0,234 | 0,194 | 0,236 | 0,303 | 0,259 | 0,205 | 0,399 | 0,391 | 0,346 | 0,305 | 0,333 | 0,289 | 0,446 |
| PCL19 | 0,305 | 0,290 | 0,396 | 0,274 | 0,375 | 0,277 | 0,261 | 0,353 | 0,345 | 0,332 | 0,393 | 0,340 | 0,268 | 0,371 | 0,286 | 0,295 | 0,640 | 0,342 | 0,241 | 0,372 | 0,406 | 0,421 | 0,383 | 0,364 | 0,281 | 0,363 |
| PCL20 | 0,270 | 0,259 | 0,236 | 0,253 | 0,236 | 0,236 | 0,286 | 0,253 | 0,270 | 0,189 | 0,251 | 0,274 | 0,670 | 0,334 | 0,252 | 0,213 | 0,261 | 0,142 | 0,149 | 0,227 | 0,318 | 0,290 | 0,414 | 0,234 | 0,164 | 0,178 |
| RTQ1 | 1,000 | 0,555 | 0,478 | 0,501 | 0,497 | 0,491 | 0,495 | 0,437 | 0,487 | 0,496 | 0,380 | 0,513 | 0,304 | 0,382 | 0,303 | 0,622 | 0,330 | 0,194 | 0,360 | 0,435 | 0,451 | 0,476 | 0,395 | 0,278 | 0,328 | 0,400 |
| RTQ2 | 0,555 | 1,000 | 0,417 | 0,715 | 0,574 | 0,604 | 0,693 | 0,531 | 0,613 | 0,577 | 0,295 | 0,396 | 0,301 | 0,368 | 0,335 | 0,455 | 0,344 | 0,217 | 0,307 | 0,396 | 0,466 | 0,465 | 0,369 | 0,294 | 0,296 | 0,388 |
| RTQ3 | 0,478 | 0,417 | 1,000 | 0,401 | 0,506 | 0,428 | 0,446 | 0,481 | 0,437 | 0,417 | 0,414 | 0,445 | 0,252 | 0,377 | 0,334 | 0,456 | 0,399 | 0,311 | 0,371 | 0,406 | 0,469 | 0,433 | 0,407 | 0,324 | 0,310 | 0,438 |
| RTQ4 | 0,501 | 0,715 | 0,401 | 1,000 | 0,583 | 0,622 | 0,677 | 0,515 | 0,613 | 0,550 | 0,261 | 0,355 | 0,289 | 0,303 | 0,281 | 0,385 | 0,284 | 0,205 | 0,260 | 0,334 | 0,387 | 0,387 | 0,329 | 0,263 | 0,242 | 0,321 |
| RTQ5 | 0,497 | 0,574 | 0,506 | 0,583 | 1,000 | 0,592 | 0,574 | 0,647 | 0,668 | 0,535 | 0,364 | 0,398 | 0,288 | 0,358 | 0,351 | 0,456 | 0,415 | 0,275 | 0,349 | 0,445 | 0,558 | 0,505 | 0,416 | 0,351 | 0,369 | 0,435 |
| RTQ6 | 0,491 | 0,604 | 0,428 | 0,622 | 0,592 | 1,000 | 0,653 | 0,554 | 0,640 | 0,587 | 0,274 | 0,374 | 0,283 | 0,300 | 0,277 | 0,383 | 0,340 | 0,167 | 0,254 | 0,423 | 0,420 | 0,427 | 0,356 | 0,256 | 0,269 | 0,387 |
| RTQ7 | 0,495 | 0,693 | 0,446 | 0,677 | 0,574 | 0,653 | 1,000 | 0,563 | 0,650 | 0,616 | 0,297 | 0,339 | 0,304 | 0,333 | 0,274 | 0,376 | 0,326 | 0,176 | 0,258 | 0,386 | 0,412 | 0,442 | 0,383 | 0,261 | 0,266 | 0,424 |
| RTQ8 | 0,437 | 0,531 | 0,481 | 0,515 | 0,647 | 0,554 | 0,563 | 1,000 | 0,698 | 0,576 | 0,353 | 0,403 | 0,284 | 0,294 | 0,325 | 0,402 | 0,373 | 0,304 | 0,377 | 0,418 | 0,516 | 0,468 | 0,417 | 0,363 | 0,338 | 0,443 |
| RTQ9 | 0,487 | 0,613 | 0,437 | 0,613 | 0,668 | 0,640 | 0,650 | 0,698 | 1,000 | 0,637 | 0,321 | 0,404 | 0,286 | 0,346 | 0,314 | 0,448 | 0,382 | 0,195 | 0,278 | 0,406 | 0,492 | 0,476 | 0,406 | 0,343 | 0,334 | 0,416 |
| RTQ10 | 0,496 | 0,577 | 0,417 | 0,550 | 0,535 | 0,587 | 0,616 | 0,576 | 0,637 | 1,000 | 0,312 | 0,398 | 0,264 | 0,316 | 0,327 | 0,412 | 0,358 | 0,229 | 0,280 | 0,377 | 0,423 | 0,394 | 0,387 | 0,289 | 0,255 | 0,414 |
| RHQ1 | 0,380 | 0,295 | 0,414 | 0,261 | 0,364 | 0,274 | 0,297 | 0,353 | 0,321 | 0,312 | 1,000 | 0,679 | 0,367 | 0,552 | 0,431 | 0,494 | 0,472 | 0,320 | 0,410 | 0,384 | 0,407 | 0,421 | 0,457 | 0,359 | 0,367 | 0,407 |
| PHQ2 | 0,513 | 0,396 | 0,445 | 0,355 | 0,398 | 0,374 | 0,339 | 0,403 | 0,404 | 0,398 | 0,679 | 1,000 | 0,373 | 0,558 | 0,412 | 0,650 | 0,419 | 0,296 | 0,512 | 0,486 | 0,534 | 0,518 | 0,495 | 0,351 | 0,381 | 0,475 |
| PHQ3 | 0,304 | 0,301 | 0,252 | 0,289 | 0,288 | 0,283 | 0,304 | 0,284 | 0,286 | 0,264 | 0,367 | 0,373 | 1,000 | 0,513 | 0,365 | 0,311 | 0,349 | 0,211 | 0,229 | 0,347 | 0,366 | 0,339 | 0,470 | 0,281 | 0,281 | 0,268 |
| PHQ4 | 0,382 | 0,368 | 0,377 | 0,303 | 0,358 | 0,300 | 0,333 | 0,294 | 0,346 | 0,316 | 0,552 | 0,558 | 0,513 | 1,000 | 0,474 | 0,465 | 0,410 | 0,254 | 0,266 | 0,460 | 0,453 | 0,473 | 0,500 | 0,274 | 0,375 | 0,370 |
| PHQ5 | 0,303 | 0,335 | 0,334 | 0,281 | 0,351 | 0,277 | 0,274 | 0,325 | 0,314 | 0,327 | 0,431 | 0,412 | 0,365 | 0,474 | 1,000 | 0,414 | 0,398 | 0,306 | 0,227 | 0,366 | 0,376 | 0,375 | 0,424 | 0,373 | 0,289 | 0,330 |
| PHQ6 | 0,622 | 0,455 | 0,456 | 0,385 | 0,456 | 0,383 | 0,376 | 0,402 | 0,448 | 0,412 | 0,494 | 0,650 | 0,311 | 0,465 | 0,414 | 1,000 | 0,391 | 0,225 | 0,472 | 0,452 | 0,517 | 0,508 | 0,400 | 0,297 | 0,328 | 0,470 |
| PHQ7 | 0,330 | 0,344 | 0,399 | 0,284 | 0,415 | 0,340 | 0,326 | 0,373 | 0,382 | 0,358 | 0,472 | 0,419 | 0,349 | 0,410 | 0,398 | 0,391 | 1,000 | 0,401 | 0,312 | 0,426 | 0,437 | 0,449 | 0,436 | 0,435 | 0,361 | 0,433 |
| PHQ8 | 0,194 | 0,217 | 0,311 | 0,205 | 0,275 | 0,167 | 0,176 | 0,304 | 0,195 | 0,229 | 0,320 | 0,296 | 0,211 | 0,254 | 0,306 | 0,225 | 0,401 | 1,000 | 0,346 | 0,256 | 0,308 | 0,270 | 0,303 | 0,491 | 0,268 | 0,260 |
| PHQ9 | 0,360 | 0,307 | 0,371 | 0,260 | 0,349 | 0,254 | 0,258 | 0,377 | 0,278 | 0,280 | 0,410 | 0,512 | 0,229 | 0,266 | 0,227 | 0,472 | 0,312 | 0,346 | 1,000 | 0,310 | 0,341 | 0,300 | 0,295 | 0,293 | 0,302 | 0,366 |
| GAD1 | 0,435 | 0,396 | 0,406 | 0,334 | 0,445 | 0,423 | 0,386 | 0,418 | 0,406 | 0,377 | 0,384 | 0,486 | 0,347 | 0,460 | 0,366 | 0,452 | 0,426 | 0,256 | 0,310 | 1,000 | 0,696 | 0,668 | 0,603 | 0,416 | 0,451 | 0,607 |
| GAD2 | 0,451 | 0,466 | 0,469 | 0,387 | 0,558 | 0,420 | 0,412 | 0,516 | 0,492 | 0,423 | 0,407 | 0,534 | 0,366 | 0,453 | 0,376 | 0,517 | 0,437 | 0,308 | 0,341 | 0,696 | 1,000 | 0,811 | 0,600 | 0,463 | 0,505 | 0,612 |
| GAD3 | 0,476 | 0,465 | 0,433 | 0,387 | 0,505 | 0,427 | 0,442 | 0,468 | 0,476 | 0,394 | 0,421 | 0,518 | 0,339 | 0,473 | 0,375 | 0,508 | 0,449 | 0,270 | 0,300 | 0,668 | 0,811 | 1,000 | 0,582 | 0,433 | 0,506 | 0,595 |
| GAD4 | 0,395 | 0,369 | 0,407 | 0,329 | 0,416 | 0,356 | 0,383 | 0,417 | 0,406 | 0,387 | 0,457 | 0,495 | 0,470 | 0,500 | 0,424 | 0,400 | 0,436 | 0,303 | 0,295 | 0,603 | 0,600 | 0,582 | 1,000 | 0,516 | 0,448 | 0,471 |
| GAD5 | 0,278 | 0,294 | 0,324 | 0,263 | 0,351 | 0,256 | 0,261 | 0,363 | 0,343 | 0,289 | 0,359 | 0,351 | 0,281 | 0,274 | 0,373 | 0,297 | 0,435 | 0,491 | 0,293 | 0,416 | 0,463 | 0,433 | 0,516 | 1,000 | 0,413 | 0,416 |
| GAD6 | 0,328 | 0,296 | 0,310 | 0,242 | 0,369 | 0,269 | 0,266 | 0,338 | 0,334 | 0,255 | 0,367 | 0,381 | 0,281 | 0,375 | 0,289 | 0,328 | 0,361 | 0,268 | 0,302 | 0,451 | 0,505 | 0,506 | 0,448 | 0,413 | 1,000 | 0,460 |
| GAD7 | 0,400 | 0,388 | 0,438 | 0,321 | 0,435 | 0,387 | 0,424 | 0,443 | 0,416 | 0,414 | 0,407 | 0,475 | 0,268 | 0,370 | 0,330 | 0,470 | 0,433 | 0,260 | 0,366 | 0,607 | 0,612 | 0,595 | 0,471 | 0,416 | 0,460 | 1,000 |

**Supplementary Figure 1.** Bootstrap edge weights difference test of the PTSD symptom clusters and rumination depicted in Figure 1.


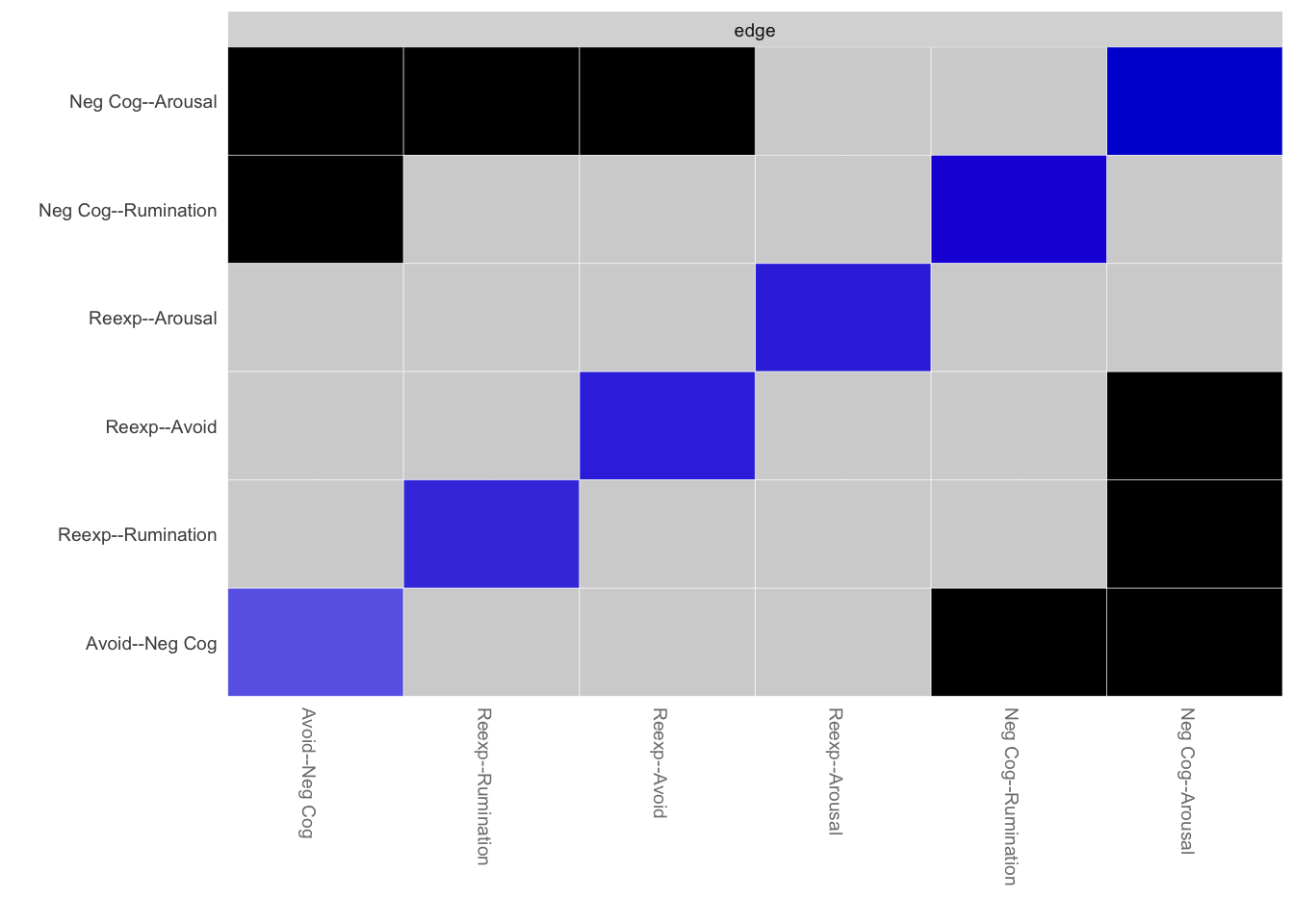


*Note*. Grey boxes indicate non-significant differences between nonzero estimated edges. Black boxes represent significant differences. Each edge´s magnitude is indicated by the saturation of colour of the diagonal boxes corresponding to the magnitude of the edges (negative to positive).

**Supplementary Figure 2.** Bootstrap 95% confidence intervals of the edge weights of the PTSD symptom clusters and rumination depicted in Figure 1.


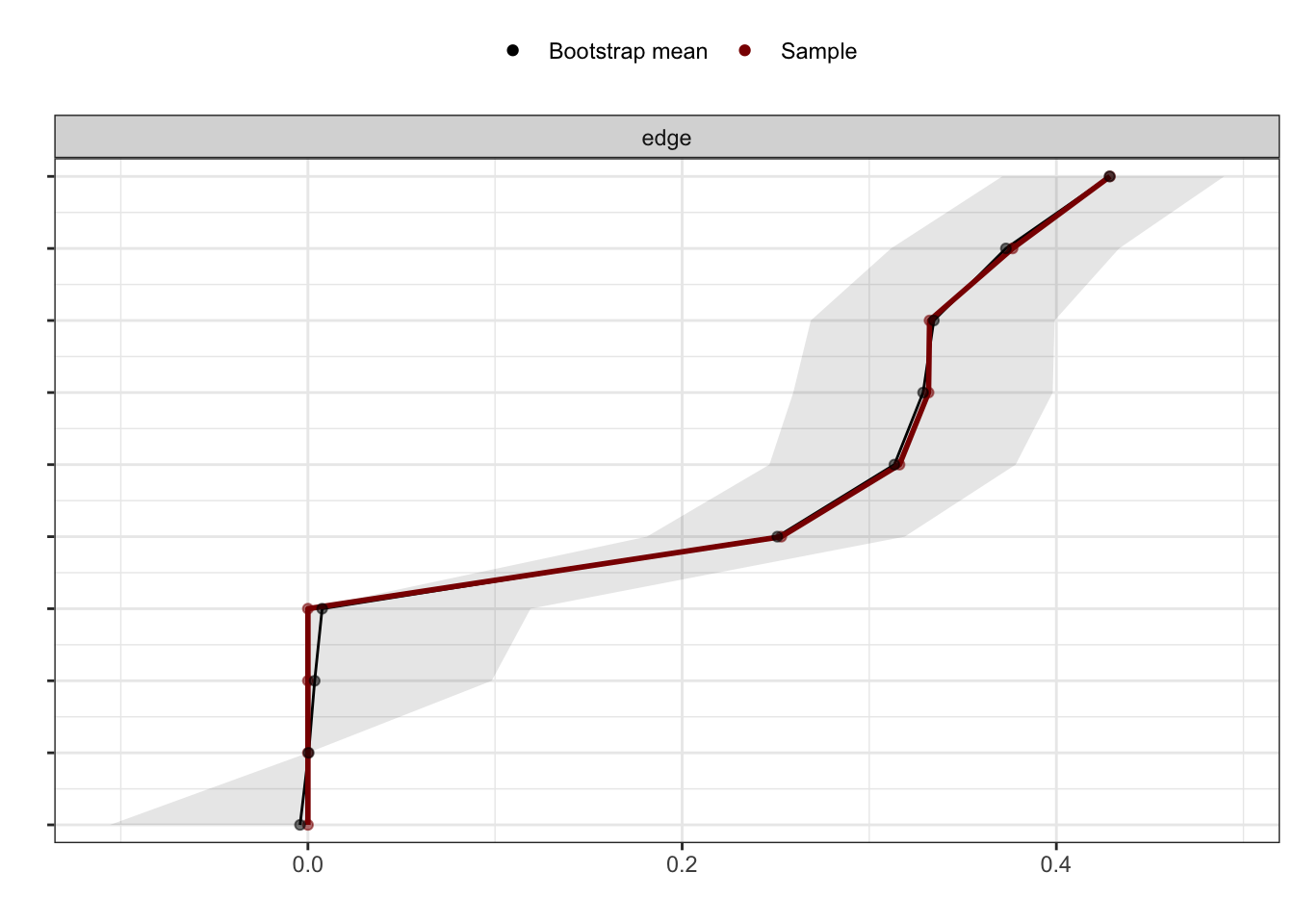


*Note.* The horizontal grey line represents individual edges, their weight is indicated by the red line. The grey area represents the bootstrap 95% confidence interval.

**Supplementary Figure 3.** Bootstrap 95% confidence intervals of the edge weights of the PTSD symptom clusters and rumination depicted in Figure 1.


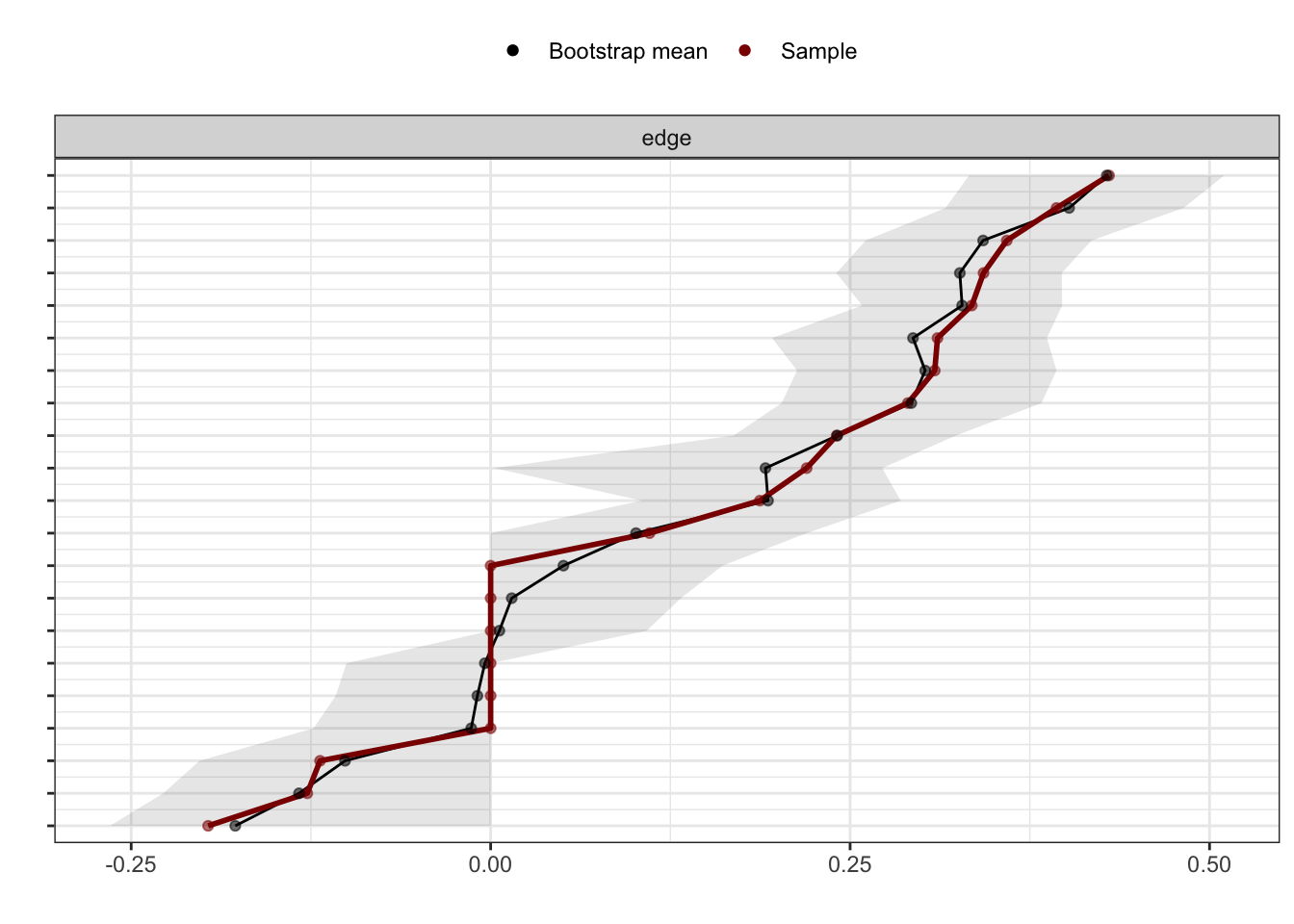


*Note.* The horizontal grey line represents individual edges, their weight is indicated by the red line. The grey area represents the bootstrap 95% confidence interval.

**Supplementary Figure 4.** Bootstrap 95% confidence intervals of the edge weights of the PTSD symptom clusters and rumination depicted in Figure 1.


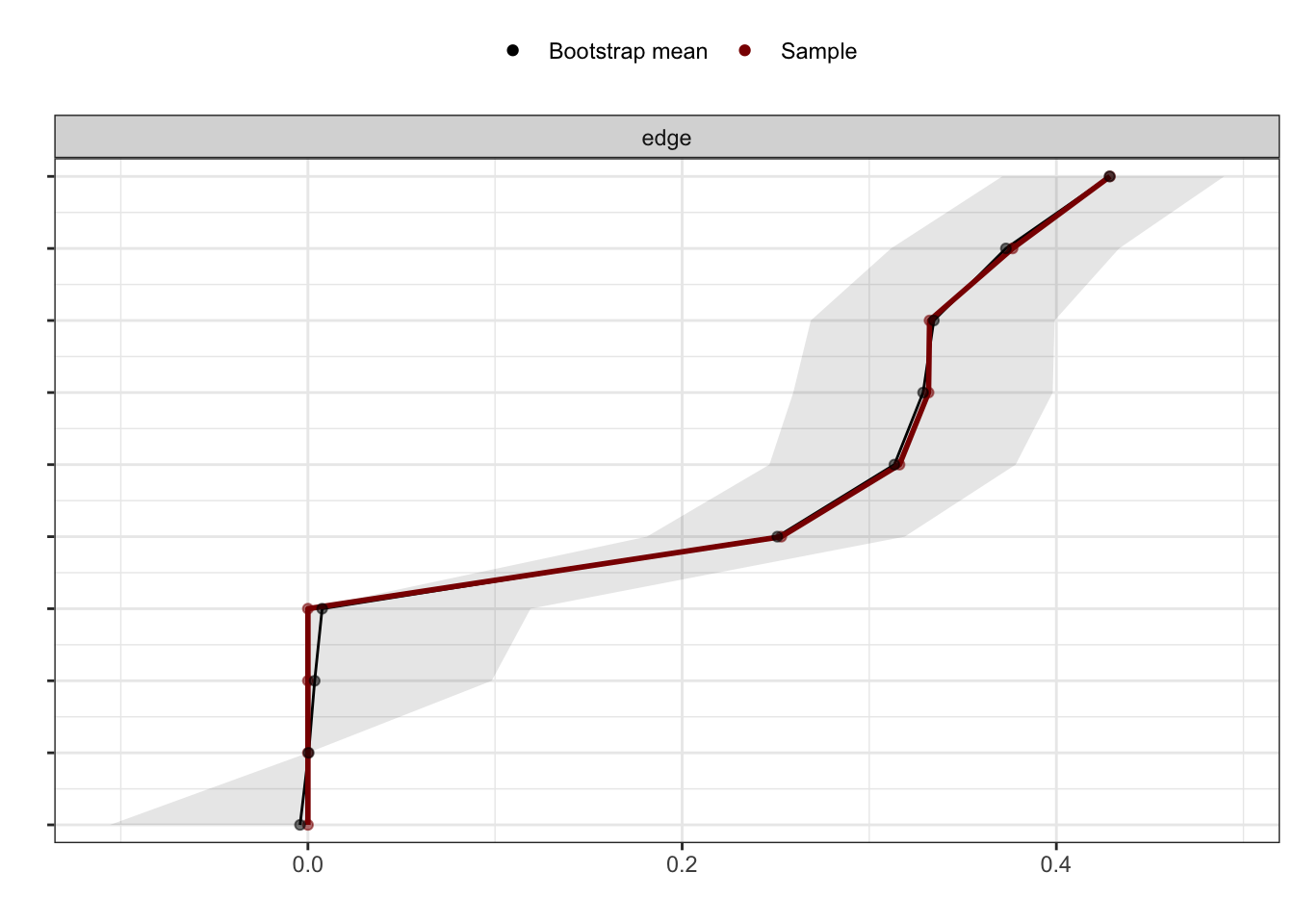


*Note.* The horizontal grey line represents individual edges, their weight is indicated by the red line. The grey area represents the bootstrap 95% confidence interval.

**Supplementary Figure 5.** Bootstrap edge weights difference test of PTSD symptom clusters, rumination, and level of anxiety and depression depicted in Figure 2.


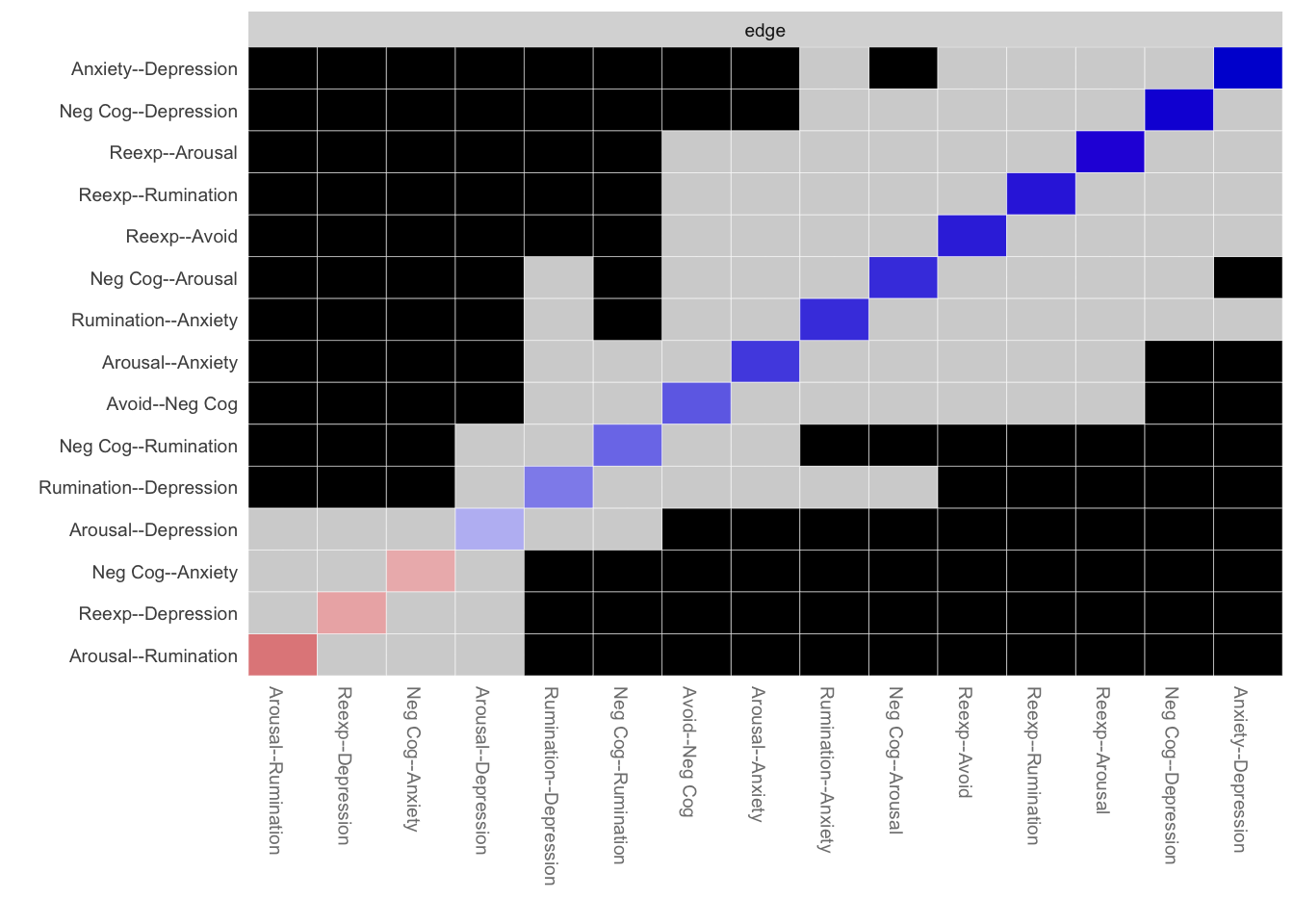


*Note*. Grey boxes indicate non-significant differences between nonzero estimated edges. Black boxes represent significant differences. Each edge’s magnitude is indicated by the saturation of colour of the diagonal boxes (ranging from red to blue) corresponding to the magnitude of the edge (negative to positive).

**Supplementary Figure 6.** Bootstrap 95% confidence intervals of the edge weights of PTSD symptom clusters, rumination, and level of anxiety and depression depicted in Figure 2.


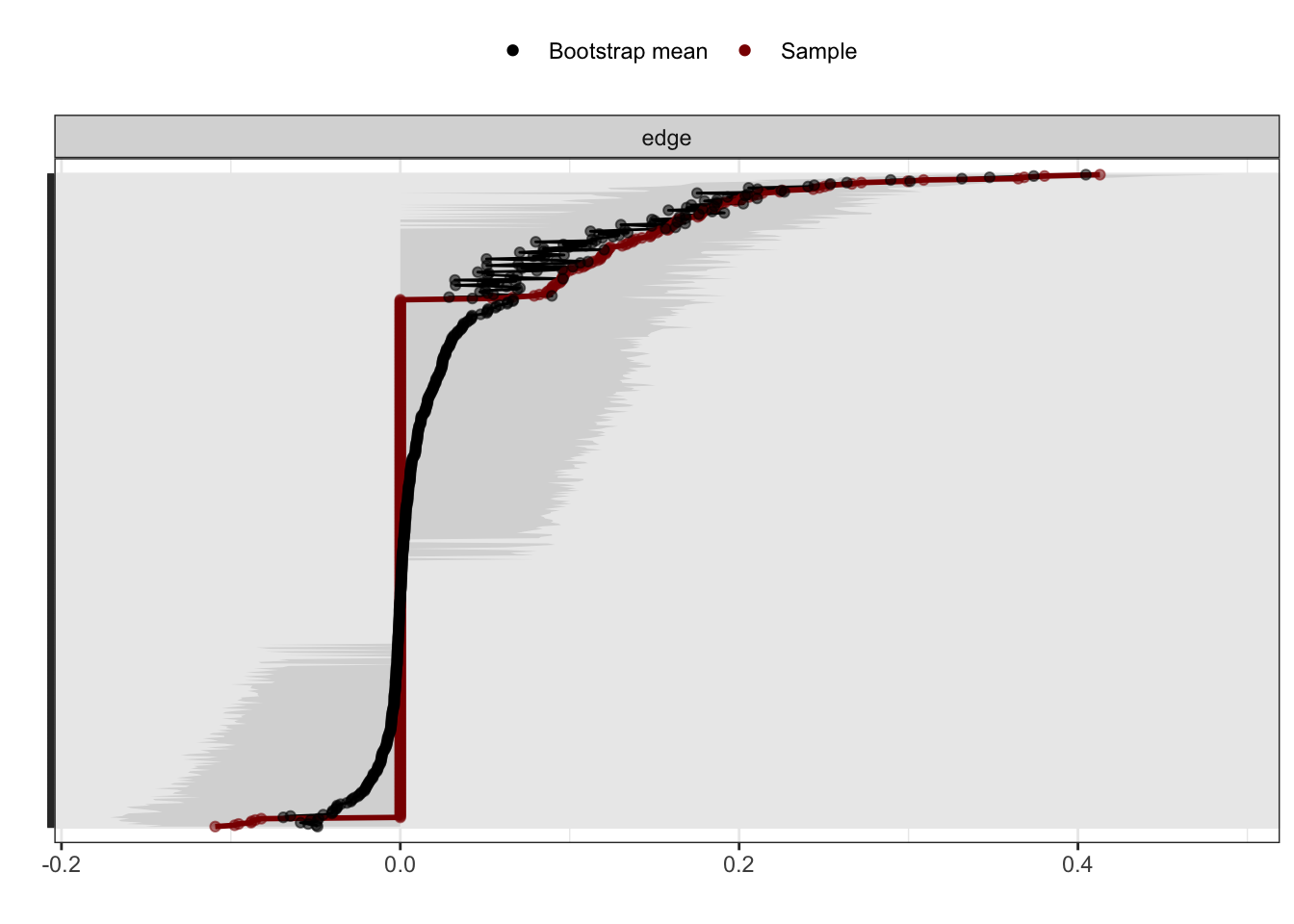


*Note*. The horizontal grey line represents individual edges, their weight is indicated by the red line. The grey area represents the bootstrap 95% confidence interval.

**Supplementary Figure 7**. Bootstrap edge weights difference test of PTSD symptoms and rumination at the item-level, adjusted for depression and anxiety levels depictured in Figure 3.


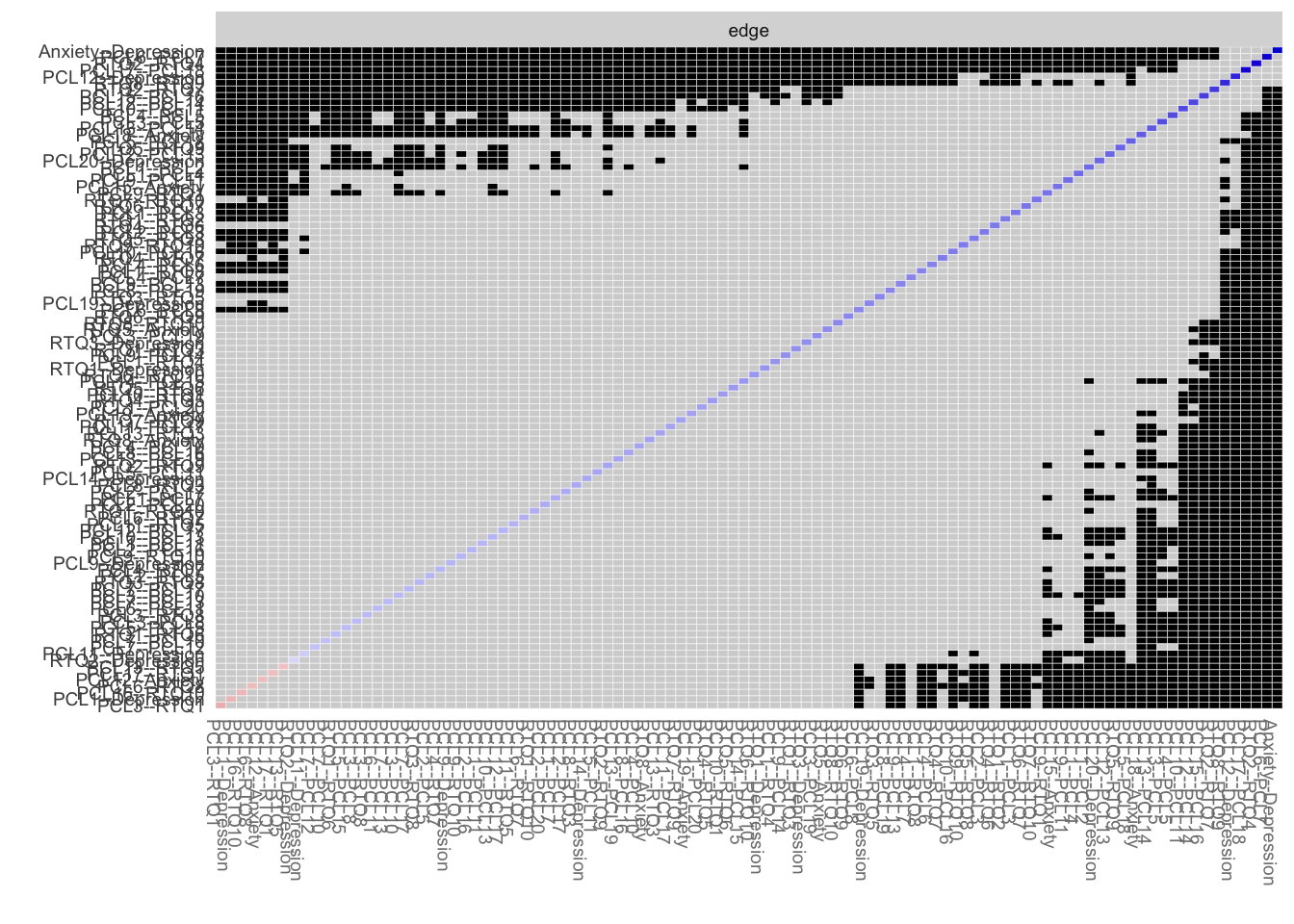


*Note*. Grey boxes indicate non-significant differences between nonzero estimated edges. Black boxes represent significant differences. Each edge’s magnitude is indicated by the saturation of colour of the diagonal boxes (ranging from red to blue) corresponding to the magnitude of the edge (negative to positive).
